# The role of an intramolecular hydrogen bond in the redox properties of carboxylic acid naphthoquinones†

**DOI:** 10.1039/d4sc05277c

**Published:** 2024-09-20

**Authors:** Walter D. Guerra, Emmanuel Odella, Kai Cui, Maxim Secor, Rodrigo E. Dominguez, Edwin J. Gonzalez, Thomas A. Moore, Sharon Hammes-Schiffer, Ana L. Moore

**Affiliations:** a School of Molecular Sciences, Arizona State University Tempe Arizona 85287-1604 USA amoore@asu.edu; b Department of Chemistry, Princeton University Princeton New Jersey 08544 USA shs566@princeton.edu

## Abstract

A bioinspired naphthoquinone model of the quinones in photosynthetic reaction centers but bearing an intramolecular hydrogen-bonded carboxylic acid has been synthesized and characterized electrochemically, spectroscopically, and computationally to provide mechanistic insight into the role of proton-coupled electron transfer (PCET) of quinone reduction in photosynthesis. The reduction potential of this construct is 370 mV more positive than the unsubstituted naphthoquinone. In addition to the reversible cyclic voltammetry, infrared spectroelectrochemistry confirms that the naphthoquinone/naphthoquinone radical anion couple is fully reversible. Calculated redox potentials agree with the experimental trends arising from the intramolecular hydrogen bond. Molecular electrostatic potentials illustrate the reversible proton transfer driving forces, and analysis of the computed vibrational spectra supports the possibility of a combination of electron transfer and PCET processes. The significance of PCET, reversibility, and redox potential management relevant to the design of artificial photosynthetic assemblies involving PCET processes is discussed.

## Abstract 2

The electrochemical properties of a bioinspired naphthoquinone bearing an intramolecular hydrogen-bonded carboxylic acid are explained by a combination of PCET and ET processes.

## Graphical abstract

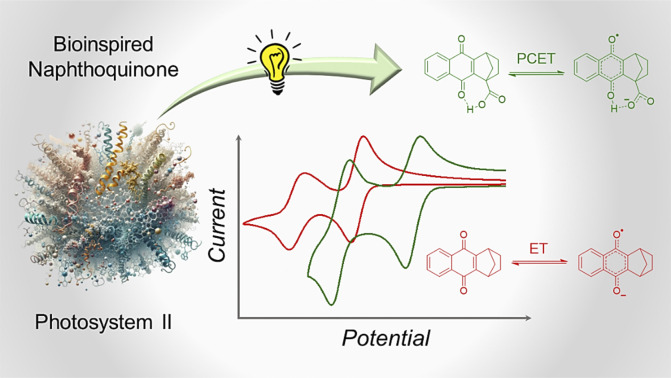

## Introduction

Quinones play an essential role as electron and proton carriers in biological processes such as respiration and photosynthesis.^1–4^ These compounds are also present in chemical oxidation methodologies,^5^ as additives in transition metal catalysis^6^ or as initiating reagents in photocatalysis.^7^ Furthermore, the redox behaviour of quinones has been explored extensively; they can be present in solution as fully oxidized (Q), single reduced (Q˙^−^), single reduced protonated (semiquinone, QH˙), or fully reduced (Q^2−^ or its protonated form quinol, QH_2_) species. The different oxidation and protonation states depend on the experimental conditions.^8–11^ In dry, neutral, aprotic media, the fully oxidized quinone usually shows two cathodic waves, which correspond to the Q/Q˙^−^ and the Q˙^−^/Q^2−^ redox couples, respectively. The intricacy of quinone properties together with their relevance in biological processes, especially bioenergetic processes such as the Q-cycle, have made the Q/QH_2_ redox couples one of the most studied models to understand biological quinonoid systems and to explore fundamental aspects of electron transfer (ET), proton transfer (PT), and proton-coupled electron transfer (PCET) processes. In the case of the latter, quinones have been the focus of a large number of studies considering the mechanism and kinetics of PCET processes and the determination of physicochemical parameters under different acid/base and reducing/oxidizing conditions.^12,13^

**Chart 1:**
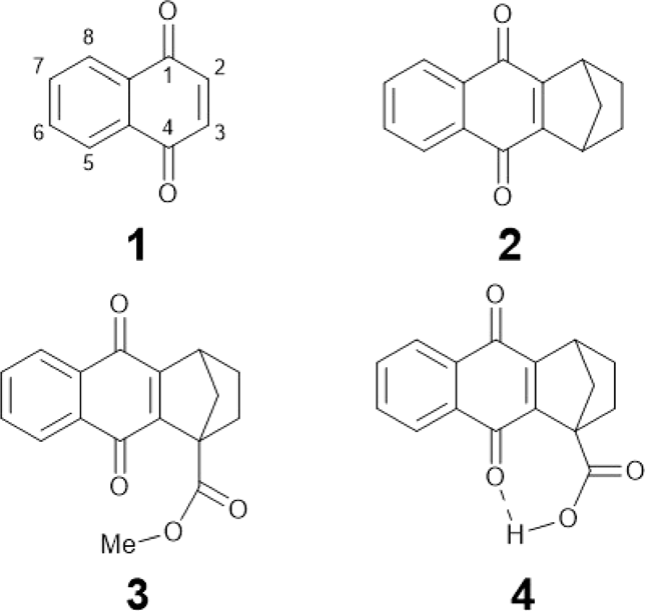
Molecular structures of NQs studied in this work: NQ (1), 2,3 substituted NQ (2), structurally related methyl ester NQ derivative (3), and NQ carboxylic acid (4).

Quinones also act as hydrogen bond acceptors,^14^ and the redox potentials of quinones are highly susceptible to the type and concentration of proton donors present in non-aqueous solvents.^9^ In particular, the effect on the electrochemical reduction process due to the intramolecular stabilization provided by hydrogen bonds was studied on phenolic quinones as models. An illustrative example is 1,4-naphthoquinone (compound 1, referred to as NQ throughout the manuscript, Chart 1) and its hydroxy derivatives (5-hydroxy-NQ and 5,8-dihydroxy-NQ), where a less negative reduction potential was observed and rationalized in terms of the OH substituent in the β position.^15,16^ In these cases, a PCET event has been proposed to explain the results. The situation for the α-hydroxy-NQ is quite different, since the typical two-monoelectronic reversible charge transfer processes are not observed.^17,18^ The proposed mechanism involves self-protonation caused by the higher acidity of the α-OH functional group. This highlights the importance of molecular structure–function relationships since the change in the position of the substituent ultimately impacts the electrochemical features. The situation with β hydroxy-anthraquinones followed the same trend observed with β hydroxy-NQs.^16^ Additionally, structural characterization of the reduced forms Q˙^−^ and/or Q^2−^ were studied by Fourier-transform infrared spectroscopy (FTIR),^19^ IR spectroelectrochemistry (IRSEC),^20^ cyclic voltabsorptometry (CVA) and derivative cyclic voltabsorptometry (DCVA), together with density functional theory (DFT) calculations. The combination of these techniques proved fruitful in the investigation into the complex electroreduction profile of quinones.^21–23^

Previous studies have also considered the electrochemical oxidation of hydroquinones to quinones (from QH_2_ to Q).^24^ These studies have extended the analysis to the 2,5-dicarboxylate-1,4-hydrobenzoquinone/2,5-dicarboxy-1,4-benzoquinone redox couple in non-aqueous medium. In this system, the role of carboxylate groups as proton accepting groups in a PCET mechanism was assigned. Kinetics together with the observation of a significant hydrogen/deuterium isotopic effect indicated that the ET and PT processes are concerted.^25^

In oxygenic photosynthesis, quinones function as electron acceptors in Photosystems I and II (PSI and PSII, respectively). These photosystems are protein complexes present in oxygen-evolving organisms where solar energy conversion takes place.^26–28^ The energy absorbed by antenna chlorophylls and related pigments is efficiently and rapidly transferred to the reaction centers. In PSII, this process is followed by charge separation and water splitting.^26^

In respiration and photosynthesis, the remarkable Q-cycle translates redox potential to proton motive force by doubling the number of protons pumped from one per electron transferred to two and thereby doubling the thermodynamic efficiency of coupling redox potential to proton motive force.^29,30^ This essential bioenergetic process remains a significant challenge for artificial systems.

Design and construction of light-harvesting antennas and artificial photosynthetic systems are key to understanding the factors that nature uses to both harvest and store solar energy.^2^ In this field, one of the fundamental approaches is to mimic the P680˙^+^–Q_A_˙^−^ charge-separated state (CSS). Several reports highlight the use of quinones as electron acceptors in chromophore-electron donor/acceptor systems,^2,31–33^ as well as in transition-metal complexes.^34–43^ It is known that hydrogen bond interactions in donor–acceptor systems perform an essential role in generating a long-lived CSS and in tuning the dynamics of the processes involved (PCET or ET).^40,44,45^ One example is the carotenoid–porphyrin–quinone triad, where the incorporation of a hydrogen-bonded carboxylic acid to the quinone moiety slows charge recombination due to the coordinated electron and proton transfer processes.^46^ In a similar fashion, some metal complexes use hydrogen bond interactions to facilitate quinone reduction either by intramolecular^40^ or intermolecular^35^ interactions.

In this work we characterize the hydrogen-bonded carboxylic acid NQ 4 (Chart 1) using electrochemistry, IRSEC, and DFT calculations to understand the reduction mechanism involved and to highlight the proposed PCET and ET mechanisms achieved by introducing the hydrogen-bonded carboxylic acid group. For comparative reasons we have synthesized two other NQ analogues, compounds 2 and 3 (Chart 1), without the carboxylic acid moiety (2) and with the methyl ester derivative (3).

## Results and discussion

### Synthesis

Compounds 1,2,3,4-tetrahydro-1,4-methanoanthracene-9,10-dione (2), as well as its methyl ester (3) and carboxylic acid (4) analogues (Chart 1), were synthesized following similar protocols previously reported.^47–51^ Synthesis and structural characterization of the NQs studied in this work are presented in Section 1.2 of the ESI† together with the full description of the synthetic intermediates in the preparation of 3 and 4. Compounds 2, 3, and 4 were synthesized with substituted 2,3 positions to avoid dimerization/addition reactions usually present in unsubstituted NQs.^9,22^ Additionally, the ester derivative 3, containing a substituent with a comparable electronic effect to the carboxylic acid of 4, was used as a reference.

### Computational characterization and electrochemistry

A theoretical and experimental characterization of the redox processes involved in these systems was performed to understand and highlight the incorporation of a hydrogen-bonded carboxylic acid to the NQ core as shown in compound 4 (Chart 1). Using DFT, the corresponding geometry optimizations and free energy calculations were performed in implicit tetrahydrofuran (THF) solution, following a protocol previously benchmarked on similar systems that yielded accurate results.^52,53^ Additional computational details are presented in Section 6 of the ESI.†

**Fig. 1:**
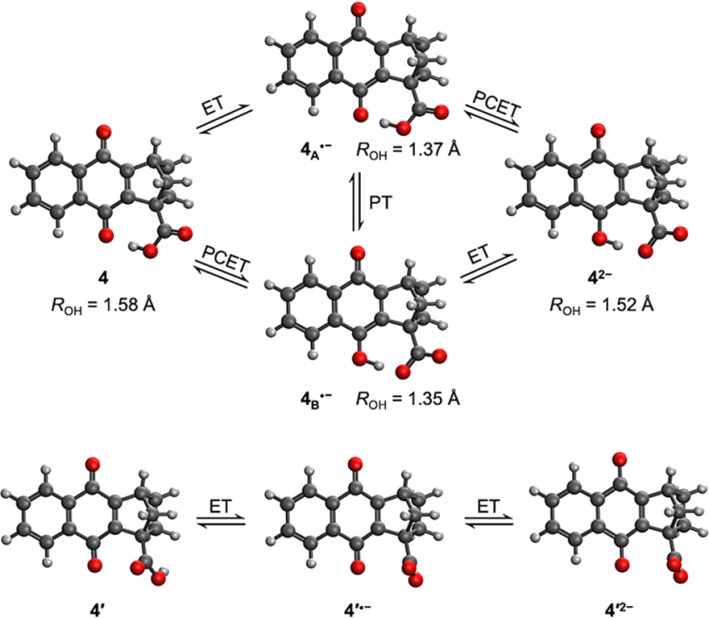
(top) Calculated structures upon electrochemical reduction of NQ 4. ET, PT, and PCET denote electron transfer, proton transfer, and proton-coupled electron transfer, respectively. Structure on the left side (4) is the neutral form, where the proton is located on the carboxylic acid. Structures 4_A_˙^−^ and 4_B_˙^−^ have been single reduced without or with intramolecular PT, respectively, corresponding to ET and PCET, respectively. The structure on the right corresponds to the double reduced species (4^2−^), the creation of which is accompanied by PT if it has not already occurred. The distance between the hydrogen and the acceptor in the hydrogen bond is denoted by *R*_OH_. (bottom) Calculated structures upon electrochemical reduction of a conformer of NQ 4, denoted 4′, which does not contain an intramolecular hydrogen bond. For 4′, both the first and second reduction steps are ET processes. In 4′˙^−^ and 4′^2−^, the proton in the carboxylic acid is behind the oxygen and thus is not visible.

The calculated structure for neutral NQ 4 (Fig. 1 top left) exhibits a hydrogen bond interaction between one of the oxygens of the carbonyl of the NQ core and the hydrogen of the carboxylic acid in the bridgehead position, indicated by the distance of *R*_OH_ = 1.58 Å in the minimum energy optimized structure. Herein, *R*_OH_ is defined as the distance between the hydrogen and its acceptor in a hydrogen-bonding interaction, which in this case is the distance between the proton in the carboxylic acid and the carbonyl oxygen in NQ 4. The hydrogen-bonded conformer is 5.5 kcal mol^−1^ more stable than the conformer without a hydrogen bond, denoted as 4′ (Fig. 1 bottom), indicating a strong hydrogen bond despite the seven-member ring.

Upon electrochemical reduction of 4, the single reduced state has two thermally accessible isomers with significant Boltzmann population, denoted 4_A_˙^−^ and 4_B_˙^−^ in Fig. 1. One possibility is that the radical anion could have a single reduced NQ core with the hydrogen-bonded carboxylic acid, forming (NQ)˙^−^–H–O–C(O), denoted 4_A_˙^−^. The *R*_OH_ distance is shortened from 1.58 Å to 1.37 Å, indicating a stronger hydrogen-bonding interaction due to the reduction. This process corresponds to pure ET. Alternatively, upon electroreduction, the single reduced protonated semiquinone, hydrogen bonded to the carboxylate anion, could be formed, generating (NQH)˙–^−^(O–C(O)), denoted 4_B_˙^−^. This process corresponds to PCET. In addition, 4_A_˙^−^ could be formed first *via* ET and then undergo a PT process to yield 4_B_˙^−^. Thermodynamically, 4_A_˙^−^ and 4_B_˙^−^ are nearly isoenergic, where 4_A_˙^−^ is only 0.29 kcal mol^−1^ more favourable. Such a difference is less than the uncertainties associated with DFT. The similar free energies of 4_A_˙^−^ and 4_B_˙^−^ are consistent with the similarity of the structural parameters: the O–H bond lengths are 1.08 Å in both 4_A_˙^−^ and 4_B_˙^−^, and the *R*_OH_ distances are 1.37 and 1.35 Å in 4_A_˙^−^ and 4_B_˙^−^, respectively. The hydrogen bond strength was calculated to be 12.6 and 16.3 kcal mol^−1^ for 4_A_˙^−^ and 4_B_˙^−^, respectively, by comparing the free energies of the conformers with an intramolecular hydrogen bond to those of conformers without this hydrogen bond (Fig. S33†). Both are significantly strengthened compared to the hydrogen bond in neutral 4 (5.5 kcal mol^−1^). Interestingly, the covalent O–H bond in NQH˙^−^ in 4_B_˙^−^ is ∼4.0 kcal mol^−1^ weaker than the covalent O–H bond in COOH in 4_A_˙^−^, based on the relative free energies of these protonation states in conformers without an intramolecular hydrogen bond (Fig. S33†). The differences in hydrogen bond strength and covalent O–H bond strength nearly cancel, resulting in similar free energies. A full thermodynamic cycle of this decomposition analysis is provided in Fig. S33 in the ESI.†

The second reduction process generates the dianion 4^2−^ (Fig. 1). Only one minimum energy geometry containing an intramolecular hydrogen bond was found with DFT for 4^2−^. This dianion could be formed either following a PCET process from 4_A_˙^−^ or an ET step from 4_B_˙^−^. In contrast to the first reduction, the second reduction weakens the hydrogen-bonding interaction but strengthens the covalent O–H bond in NQH^2−^. Specifically, comparing the geometrical changes from 4_B_˙^−^ to 4^2−^, the covalent O–H bond length decreases from 1.08 Å to 1.01 Å, while the *R*_OH_ distance increases from 1.35 Å to 1.52 Å. As shown in Fig. S33,† the calculated hydrogen bond strength in 4^2−^ is 10.3 kcal mol^−1^, which is stronger than that in neutral 4 but weaker than those in 4_A_˙^−^ and 4_B_˙^−^. However, the covalent O–H bond in NQH^2−^ is ∼15.8 kcal mol^−1^ stronger than the covalent O–H bond in the COOH group of 4′^2−^, based on the relative free energies of these protonation states in conformers without an intramolecular hydrogen bond (Fig. S33†). Assuming that the covalent O–H bond strength in COOH is not affected by the reduction of NQ, we can then conclude that the covalent O–H bond is strengthened by ∼19.8 kcal mol^−1^ in 4^2−^ compared to NQH˙^−^ in 4_B_˙^−^. Both the hydrogen bond strength and the covalent O–H bond strength correlate well with the relevant distances at the hydrogen-bonding interface.

Fig. 1 (bottom) also depicts the reduction process of 4′, which does not contain an intramolecular hydrogen bond. Due to the 5.5 kcal mol^−1^ free energy difference between 4 and 4′, the mole fraction of 4′ is negligible at room temperature. The conformer 4′ thus has no contribution to any experimentally observed phenomena but can be used to illustrate the influence of the intramolecular hydrogen bond on the reduction potentials, as discussed below.

**Fig. 2:**
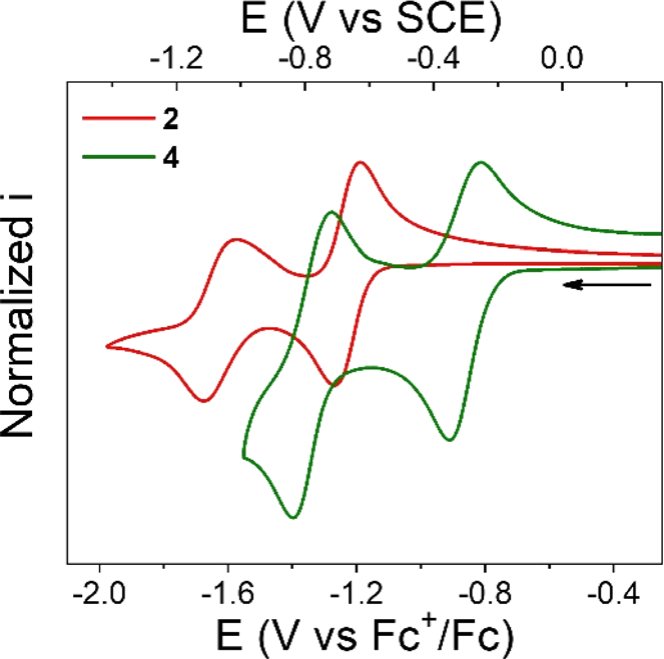
Normalized cyclic voltammograms of 2 (red line) and 4 (green line). Experimental conditions: 1 mM of 2 or 4, 0.1 M TBAPF_6_ supporting electrolyte in degassed THF. WE: glassy carbon. Pseudo RE: Ag wire (ferrocene as internal reference). CE: Pt wire. Scan rate, 100 mV s^−1^.

Cyclic voltammetry (CV) of NQs 1–4 was performed in degassed THF solution using tetrabutylammonium hexafluorophosphate (TBAPF_6_, 0.1 M) as the supporting electrolyte. The midpoint potentials (*E*_1/2_) for all the compounds were determined as the average of the cathodic and anodic peak potentials (*E*_1/2_ = (*E*p_c_ + *E*p_a_)/2) and are summarized in Table 1. Fig. 2 shows the CVs of compounds 2 and 4, while the CVs of 1 and 3 are shown in Fig. S28 and S29 in the ESI,† respectively.

**Table 1:** Electrochemical data of compounds 1–4 in THF

Compound	*E*_1/2_ a/V *vs.* Fc^+/0^ (Δ*E*_p_^b^/mV)			
	Experimental NQ/NQ˙^−^	Calculated NQ/NQ˙^−^	Experimental NQ˙^−^/NQ^2−^	Calculated NQ˙^−^/NQ^2−^
1	−1.19 (60)	−1.27	−1.64 (80)	−1.71
2	−1.23 (70)	−1.37	−1.62 (110)	−1.77
3	−1.23 (300)	−1.23^c^	−1.64 (310) P_2_^d^	−1.64^c^
			−1.85 (130) P_3_^d^	
4	−0.86 (100)	−0.92 (4_A_˙^−^)	−1.34 (120)	−1.06 (4_A_˙^−^)
		−0.93 (4_B_˙^−^)		−1.04 (4_B_˙^−^)
4′	N/A	−1.22	N/A	−1.64

a Midpoint potential *vs.* Fc^+/0^ in degassed THF, 0.1 M TBAPF_6_.

b Δ*E*_p_ measured at *ν* = 100 mV s^−1^.

c Reference compound for the computed values in this table; calculated redox potential agrees with experiment by construction.

d P_2_ and P_3_ are the estimated midpoint potentials of the two redox couples in the region of NQ˙^−^/NQ^2−^ (see Fig. S29).

Compound 2 exhibits two, one-electron reduction processes (Table 1 and Fig. 2), with the *E*_1/2_ for the NQ/NQ˙^−^ and NQ˙^−^/NQ^2−^ redox couples of −1.23 and −1.62 V *vs.* Fc^+/0^, respectively. These values are close to the redox potentials measured for 1. The only difference is that the first reduction process in 2 is 40 mV more negative than that in 1. This could be interpreted in terms of the electron donating nature of the alkyl substitution of the bicycle (2,3 positions of the NQ core).

Compound 3 presents a first, quasi reversible one-electron reduction process with *E*_1/2_ of −1.23 V *vs.* Fc^+/0^, like the NQ/NQ˙^−^ redox couple observed in compound 2. The subsequent reduction process in 3 is irreversible with estimated *E*_1/2_ of −1.64 V *vs.* Fc^+/0^, similar to that determined for 2. This observation suggests a minimal electronic effect by the COOMe group on the NQ core electrochemistry.

In contrast, the first redox couple for 4 occurs at −0.86 V *vs.* Fc^+/0^, a considerably higher potential compared to that for its analogue without the carboxylic acid (2) or ester moiety (3). In other words, 4 is 370 mV easier to reduce than 2 or 3, presumably due to the stronger hydrogen bond in the formed radical anion, as determined computationally either with or without PT (*i.e*., for an ET or PCET mechanism, see Fig. 1). Thus, the incorporation of a hydrogen-bonded carboxylic acid in the bridgehead position of the bicycle, in positions 2,3 of the NQ core, results in a significant change in the thermodynamics of the reduction process. It is worth mentioning that the substitution with the pendant carboxylic acid does not affect the chemical reversibility, contrary to that for other NQs bearing carboxylic acids, where a more complex reduction profile and complete loss of chemical reversibility is observed.^54^ A similar anodic shift is also observed for the second redox couple. Computational characterization of the structures suggests that the double reduced form is stabilized more by a stronger covalent O–H bond in NQH^2−^ than by the intramolecular hydrogen bond (Fig. S33†).

The computationally obtained redox potentials agree with the experimental trends between compounds 1 and 4 (see Section 6 of the ESI† for computational details). The results show an increase in redox potential upon addition of the carboxylic acid moiety (Table 1). For compound 4, the intramolecular hydrogen-bonding interaction increases the computed redox potential by 450 mV for the first redox couple compared to the NQ without the carboxylic acid moiety (2). In contrast, for the conformer without the intramolecular hydrogen bond, 4′, the calculated redox potential shifts are significantly smaller, only 150 mV higher compared to 2. The calculated potentials for 4′ are very close to those for 3 with the ester substitution. This computational result strongly suggests that the experimentally observed potential shift is due to the intramolecular hydrogen bond. Both the experimental and calculated NQ˙^−^/NQ^2−^ redox potentials for 4 are less negative than the corresponding redox potentials for 1, 2, and 3, most likely because protonation of the quinone electrostatically favours reduction (see Fig. S37†). However, the calculated NQ˙^−^/NQ^2−^ redox potential is less negative than the experimental value for 4, presumably due to limitations of the DFT functional.

**Fig. 3:**
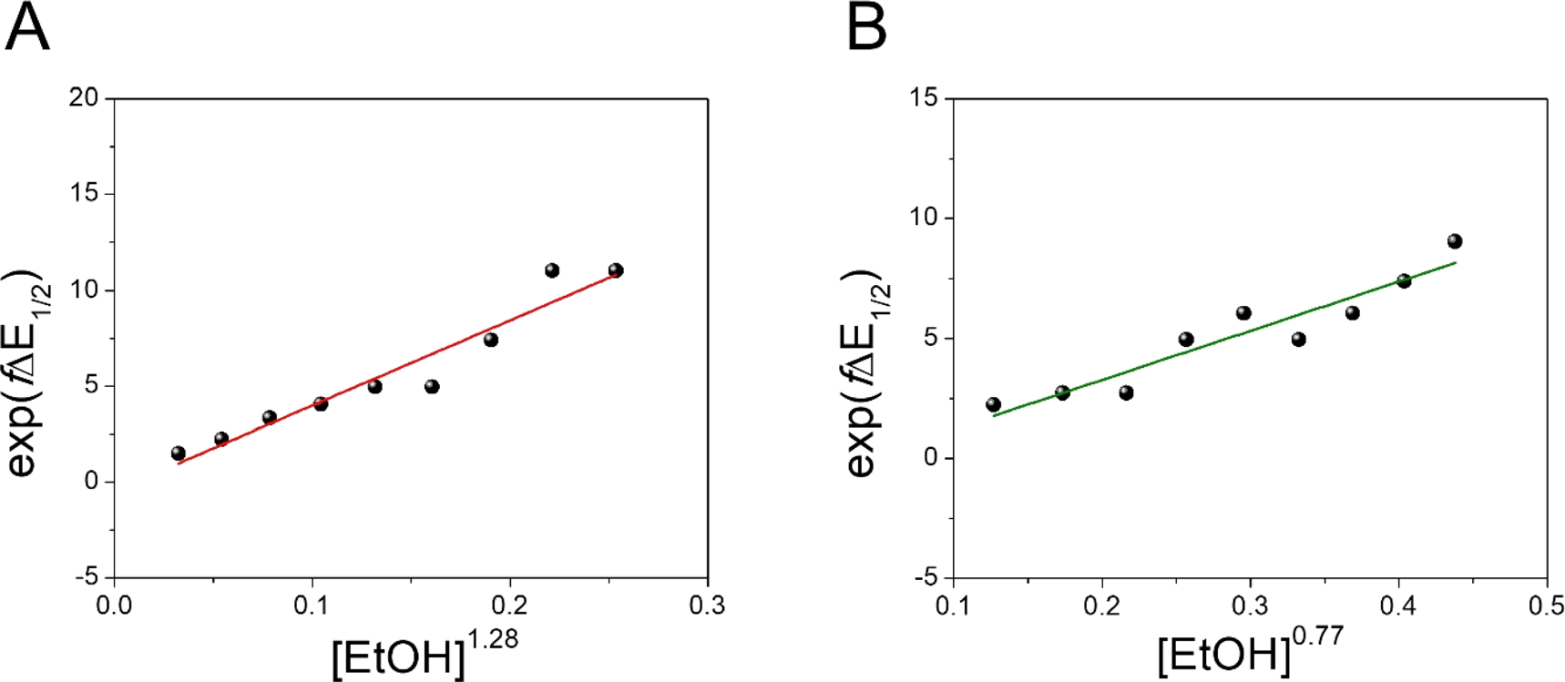
Effect of a weak hydrogen-bonding agent on the reduction processes of NQ 2 and 4. Dependence of exp(*f*Δ*E*_1/2_) *vs.* [EtOH]^*n*^ for 2 (A) and 4 (B). The equilibrium constant (*K*^(1)^_eq_) for the hydrogen bond interaction is obtained from the slope of the linear fit. Values of *n* determined for 2 and 4 are 1.28 and 0.77, respectively (see Section 3.3 in the ESI†). Complete cyclic voltammograms of 2 and 4 at different concentrations of EtOH in TFH (scan rate = 100 mV s^−1^) are presented in Fig. S30.†

Moreover, we evaluated the effect on the first reduction process by adding an external hydrogen-bonding agent to highlight the role of the intramolecular hydrogen-bonding activity of the carboxylic acid. This was done by adding increasing amounts of EtOH (considered a weak hydrogen-bonding agent)^9^ to compounds 2 and 4. The resulting changes are shown in Fig. S30 and Table S1.† The voltammetric response for 2 and 4 displays an anodic shift as the concentration of EtOH increases, with no further loss of reversibility (up to 0.34 M of EtOH). This can be rationalized by the fast stabilization of the formed radical anion through hydrogen-bonding interactions with EtOH molecules. Employing previously reported methods to quantify hydrogen-bonding constants,^55^ as well as Linschitz's treatment^9^ for the first one-electron reduction process (NQ/NQ˙^−^ redox couple), we were able to estimate the association constant (*K*_eq_^(1)^) between the hydrogen bond donor (EtOH) and the reduced NQ˙^−^ species (Fig. 3) using the following equation for the first reduction process:1
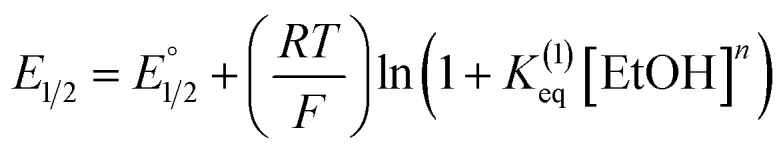
where 
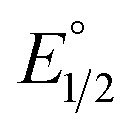
 is the midpoint potential recorded in absence of EtOH and *n* denotes the number of EtOH molecules interacting with NQ˙^−^. If *K*^(1)^_eq_[EtOH]^*n*^ ≫ 1, then a plot of *E*_1/2_*vs.* log[EtOH] should give a straight line with slope 2.3*nRT*/*F*, from which *n* can be estimated (Fig. S31, Section 3.3 in the ESI†). Rearranging eqn (1), *K*^(1)^_eq_ can be estimated as follow:2
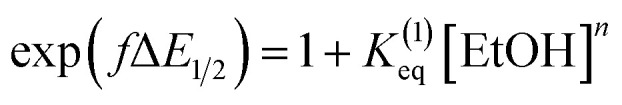
where *f* = *F*/*RT*, and 
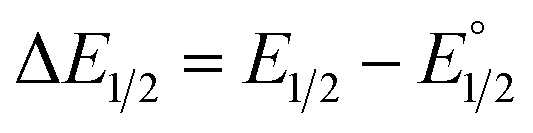
. Values of *K*^(1)^_eq_ estimated for NQs 2 and 4 were 47 and 19 M^−1^ respectively, indicating a more favourable interaction of EtOH with the NQ without the carboxylic acid (2). In the case of 4, the carboxylic group not only causes steric hindrance toward bonding with EtOH molecules, but a fraction of intermolecular hydrogen bond sites are blocked because the carboxylic acid at the bridgehead position is intramolecularly hydrogen-bonded with the NQ core, thus decreasing the interaction with the external hydrogen-bonding agent and explaining the lower *K*^(1)^_eq_ value.^9^

### Infrared spectroelectrochemistry (IRSEC) studies

IRSEC was employed to characterize the species formed *in situ* upon electrochemically reducing conditions. In both cases, reductive polarization for compounds 2 and 4 showed multiple changes, including several isosbestic points indicating that the oxidized NQ form is being progressively and reversibly converted into the reduced NQ˙^−^ form in its corresponding protonation state.

**Fig. 4:**
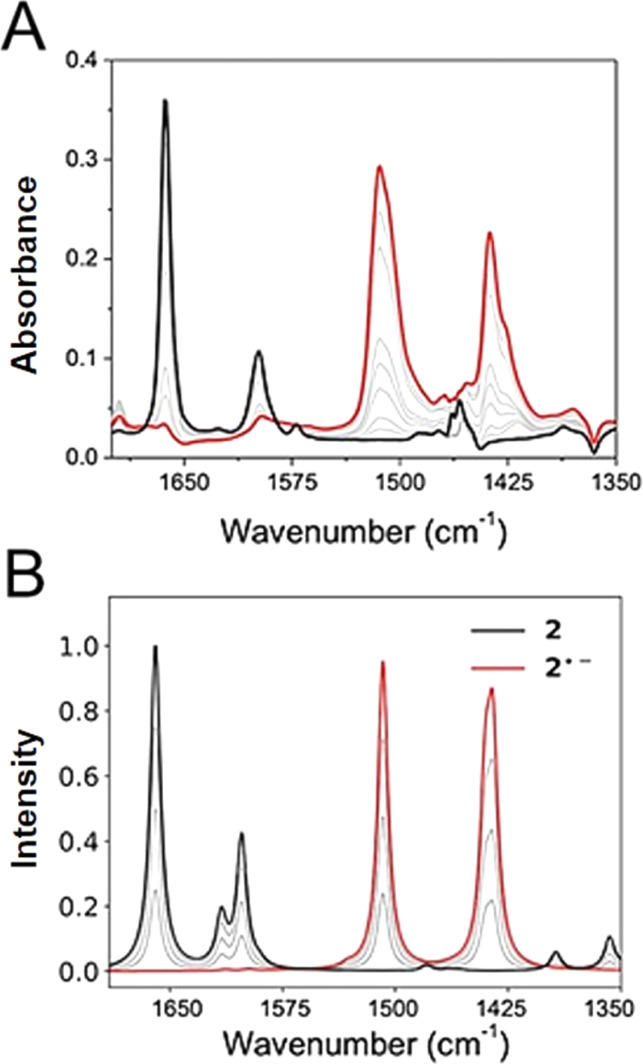
Experimental (A) and calculated (B) IRSEC spectra of 2 recorded under reductive polarization. Black traces represent the spectra of the neutral species (no polarization), red spectra show the resulting reduced species after bulk electrolysis, and grey traces display intermediate situations. Solvent: dry THF, 0.1 M TBAPF_6_. The computed frequencies were scaled by 0.983.

Experimental and computed IRSEC for 2 are depicted in Fig. 4. Electroreduction of 2 shows a decrease in the band intensity at 1663 cm^−1^ assigned to the carbonyl stretching mode (*ν*C=O), in combination with a decrease in the band intensity of an *ν*C=C mode around 1600 cm^−1^. Simultaneously, an intense and characteristic band appears at 1514 cm^−1^, which is assigned to a stretching vibration 
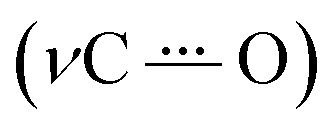
 of the radical anion 2˙^−^. This vibrational mode has been reported in the 1530–1380 cm^−1^ range for several NQs.^56–58^ These assignments were confirmed computationally (Fig. 4B), showing the disappearance of *ν*C=O and *ν*C=C bands, together with the appearance of the 
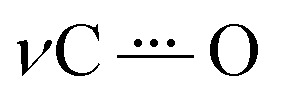
 band of 2˙^−^. Computational details can be found in Section 6 of the ESI, including Table S2,† which lists the computationally obtained frequency assignments. Calculated IRSEC spectra of the second reduction are also presented (Fig. S34†), which further corroborates the assignment of the radical anion bands (NQ˙^−^).

**Fig. 5:**
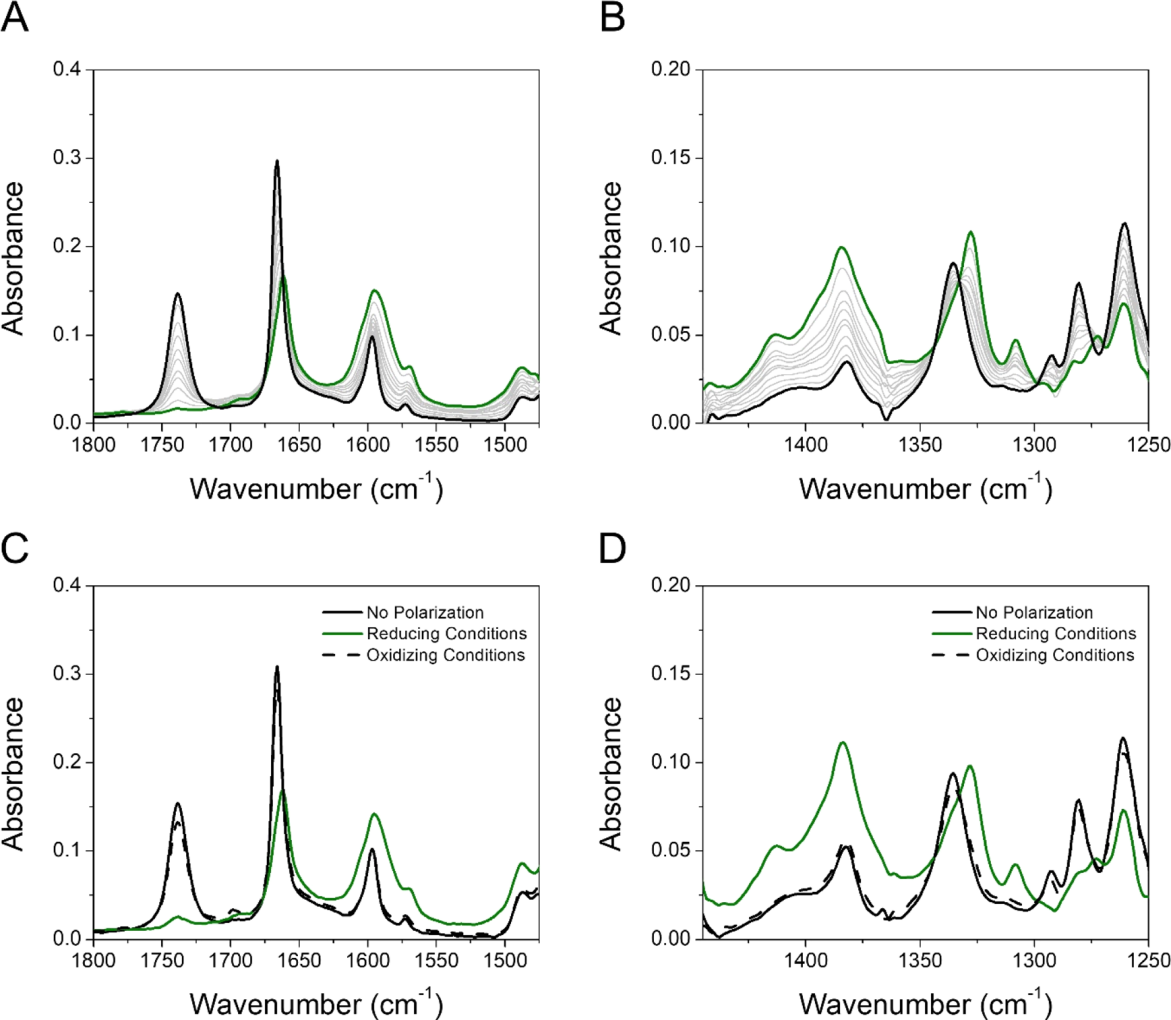
Experimental IRSEC spectra of 4 in the regions 1800–1475 cm^−1^ (A) and 1450–1250 cm^−1^ (B) recorded under reductive polarization. Reversibility assay studied in the same regions (C and D). In A and B, black solid traces represent the spectra of the neutral species (no polarization), green solid spectra show the resulting reduced species after bulk electrolysis, and grey solid traces display intermediate situations. In (C) and (D), black solid traces represent the neutral species (no polarization), green solid spectra show the resulting reduced species, and black dashed lines represent the spectrum obtained upon polarizing the solution to oxidizing potentials after reducing for 2 min at a potential required to deplete the neutral 4. Solvent: dry THF, 0.1 M TBAPF_6_.

The reductive polarization of 4 (Fig. 5) also evidenced multiple changes in regions 1800–1475 cm^−1^ (Fig. 5A) and 1450–1250 cm^−1^ (Fig. 5B). Particularly, the band at 1738 cm^−1^, assigned to the asymmetric stretching vibration of the COOH group (*ν*_as_COOH)^54,59^ disappeared upon electroreduction with the simultaneous decrease in the band intensity of *ν*C=O at 1666 cm^−1^. Concurrently, a new band appeared at around 1660 cm^−1^, which is assigned to the asymmetric stretching vibration of the C(O)O^−^ group (*ν*_as_C(O)O^−^) in concordance with literature reports.^60^ This band, in addition to the corresponding symmetrical stretching mode of the C(O)O^−^ group (*ν*_s_C(O)O^−^) at around 1380 cm^−1^, provides evidence that during the electroreduction process the carboxylic acid is being converted into its carboxylate anion (formation of 4_B_˙^−^). Additionally, the perturbation of the C–O stretching mode also indicates proton transfer upon reduction, *i.e.*, the band centered at 1335 cm^−1^ (neutral 4) decreases and the band at 1328 cm^−1^ appears (4_B_˙^−^). Other features supporting the reduction of 4 into 4_B_˙^−^ are the increase of bands associated with the *ν*C=C aromatic modes (bands at 1595 and 1569 cm^−1^). Overall, the formation of 4_B_˙^−^ can be spectroscopically tracked by the carboxylic acid/carboxylate anion conversion while the NQ form 4 is being electrochemically reduced. Particularly, the 
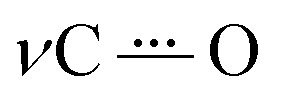
 characteristic mode is not present in the same fashion as in 2, probably due to its concurrent protonation.

Computationally, the IRSEC spectra of both the 4/4_A_˙^−^ and 4/4_B_˙^−^ redox couples were modelled (Fig. S35, Table S4†). For neutral 4, we found that the peak near 1730 cm^−1^ is the *ν*_as_COOH, and the peak near 1600 cm^−1^ consists of NQ ring modes mixed with the *ν*C=O. The main carbonyl stretch peaks of the NQ are located at 1635 and 1676 cm^−1^. In contrast, experimentally only one strong peak is observed in this region. Upon reduction, for both 4_A_˙^−^ and 4_B_˙^−^ we observed a new peak appearing at around 1550 cm^−1^. This peak is assigned to the OH bend and asymmetric stretch of the carboxylic acid/carboxylate for 4_A_˙^−^ and 4_B_˙^−^, respectively. However, other features in the simulated spectra differ significantly between 4_A_˙^−^ and 4_B_˙^−^. For example, the peak near 1400 and 1700 cm^−1^ was only observed for 4_A_˙^−^ but not for 4_B_˙^−^. Conversely, 4_B_˙^−^ exhibits a wide band consisting of multiple intense peaks between 1400 cm^−1^ to 1500 cm^−1^, but these features are absent in 4_A_˙^−^. Assignment of peaks *via* visual inspection (Table S4†) indicates that such differences arise from the different carboxylic acid/carboxylate stretch frequencies in 4_A_˙^−^ and 4_B_˙^−^, respectively. The asymmetric and symmetric stretch modes of the carboxylic acid/carboxylate were found at 1700/1562 cm^−1^ and 1404 cm^−1^ for 4_A_˙^−^, but at 1540/1414 cm^−1^ and 1483 cm^−1^ for 4_B_˙^−^. Experimentally, reduced 4 has no significant bands around 1700 cm^−1^ that might indicate the formation of 4_A_˙^−^, but several other bands at around 1660 cm^−1^ and 1400–1500 cm^−1^ could indicate the coexistence of 4_A_˙^−^ and 4_B_˙^−^. The cohabitation of both species is also consistent with the similar computed free energies of 4_A_˙^−^ and 4_B_˙^−^.

As a control experiment, we used the organic base tetrabutylammonium hydroxide (TBAOH) to identify the deprotonated form of 4 (4^−^). Adding TBAOH (5 equivalents) in a solution of 4 in THF led to a full consumption of the carboxylic acid form, as shown by the disappearance of the *ν*_as_COOH band (Fig. S32†). Additionally, the profile observed in both regions resembles the reduced species obtained upon electroreduction conditions, proving that the carboxylic proton is transferred to the carbonyl group of the quinone by a PCET process or by an ET process followed by a proton transfer process.

Furthermore, we have performed experiments switching the electrode potential to oxidative conditions. Our results show that holding the potential to oxidative conditions (Fig. 5C and D) effectively reverses the proton transfer, and the neutral 4 species is basically completely restored. This highlights both the significance of an intramolecular hydrogen bond and the electrochemical reversibility of the process.

### Computational molecular electrostatic potential studies

**Fig. 6:**
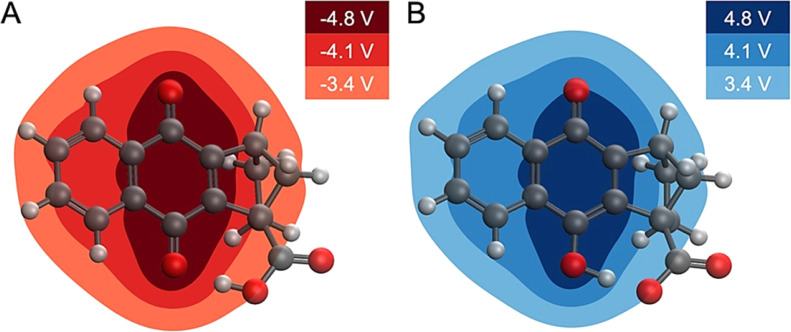
Changes in MEP upon electrochemical reduction or oxidation. (A) Change in MEP upon reduction of 4. (B) Change in MEP upon oxidation of 4_B_˙^−^, where proton transfer has occurred. The change in MEP is roughly equal and opposite for the reduction and oxidation processes inducing proton translocation. The changes in MEP are given in Volts.

Computational analysis of the molecular electrostatic potentials (MEPs) shows the electrostatic driving force of proton transfer upon electrochemical reduction and oxidation (Fig. 6). MEP analysis has proven useful in previous studies of similar systems.^61–63^ The change in MEP, Δ*V*(**r**), at a point **r** upon reduction may be expressed in atomic units as3
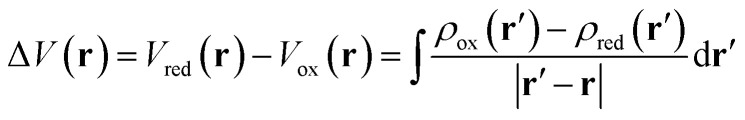
where *V*_red_(**r**) is the MEP of the molecule in the reduced state, *V*_ox_(**r**) is the MEP of the molecule in the oxidized state, *ρ*_red_(**r**) is the electronic density of the reduced state, and *ρ*_ox_(**r**) is the electronic density of the oxidized state. The form of eqn (3) after the second equality does not contain any terms involving the position of nuclei because the change in MEP is instantaneous upon electrochemical reduction, and no nuclear relaxation (including proton translocation) has yet to occur. The negative gradient of this change in MEP is the electric field experienced by the nuclei upon electrochemical reduction.

This analysis implies that an electric field drives the proton translocation across the proton transfer interface upon electrochemical reduction of 4. Fig. 6A shows the change in MEP, Δ*V*(**r**), upon the first reduction of 4. A valley of negative Δ*V*(**r**) forms around the reduction center of the quinone system (*i.e.*, the carbonyl group), and the change in the MEP decreases in magnitude across the proton transfer interface toward the carboxylic acid. This also occurs in the reverse direction upon electrochemical oxidation, with a nearly equal and opposite electrostatic driving force causing proton translocation back to the quinone (Fig. 6B). The second reduction of 4 exhibits similar behaviour (Fig. S37†). This equal and opposite driving force in reducing and oxidizing conditions is consistent with the reversibility of the overall PCET process. Computational details relevant to the calculation of the MEP plots are provided in Section 6 of the ESI.†

## Conclusions

The bioinspired hydrogen-bonded carboxylic acid NQ 4 presented in this work illustrates the effect achieved by incorporating an intramolecular hydrogen bond to the quinone core in the reduction mechanism. A remarkable increase in the reduction potentials, 370 mV in comparison to its analogue without the carboxylic acid, was observed. Additionally, titration studies were used to estimate the equilibrium constant for the hydrogen bond interaction between an external agent (EtOH) and the NQ˙^**−**^, showing that the intramolecular hydrogen bond present in 4 effectively competes with the external addition of EtOH.

IRSEC together with DFT calculations were useful to illustrate an ET/PCET-based mechanism in the electroreduction of 4. Furthermore, the reductive–oxidative polarization cycle in this compound was also demonstrated by IRSEC, and computational modeling showed that proton transfer electrostatic driving forces are similar but in the opposite directions for the reductive and oxidative ET/PCET processes. Demonstration of the reversibility accomplishes a main goal in the design of biologically-relevant, bioinspired constructs,^64^ as well as in future systems, including quinone-amino acid constructs. The substituted NQ constructs could be further adapted into novel designs of donor–acceptor systems, as well as metal complexes employed in photocatalysis, where the incorporation of ET/PCET processes could generate long-lived charge-separated states and expand the horizon of its applications.

## Author contributions

W. G. and E. O. designed the experiments and conducted them, K. C. and M. S. performed all the computational studies, R. D. and E. G. prepared and characterized some of the compounds, W. G., E. O., K. C., M. S., T. M., S. H.-S. and A. M. wrote and edited the manuscript.

## Conflicts of interest

There are no conflicts to declare.

## Supplementary Material

SC-015-D4SC05277C-s001

## Data Availability

The data supporting this article have been included as part of the ESI.†
