# Supplementary material for: The role of an intramolecular hydrogen bond in the redox properties of carboxylic acid naphthoquinones
Source: Chem Sci. 2024 Sep 20;15(42):17425–34. doi: 10.1039/d4sc05277c (PMC11445641; doi:10.1039/d4sc05277c)
Supplement: SC-015-D4SC05277C-s001 [file SC-015-D4SC05277C-s001.pdf]

Supplementary Information (SI) for Chemical Science.
This journal is © The Royal Society of Chemistry 2024

The Role of an Intramolecular Hydrogen Bond in the Redox Properties of Carboxylic Acid Naphthoquinones

Walter D. Guerra,[§] Emmanuel Odella,[§,Δ] Kai Cui,[†] Maxim Secor,[†] Rodrigo E. Dominguez,[§] Edwin J. Gonzalez,[§] Thomas A. Moore,[§] Sharon Hammes-Schiffer,[†,*] and Ana L. Moore[§,*]

§ School of Molecular Sciences, Arizona State University, Tempe, Arizona 85287-1604, United States.

† Department of Chemistry, Princeton University, Princeton, New Jersey 08544, United States.

Δ Current address: Instituto de Investigaciones en Tecnologías Energéticas y Materiales Avanzados (IITEMA), Universidad Nacional de Río Cuarto (UNRC), Consejo Nacional de Investigaciones Científicas y Tecnológicas (CONICET), Río Cuarto X5804BYA, Córdoba, Argentina.

*Corresponding authors: Sharon Hammes-Schiffer (shs566@princeton.edu) and Ana L. Moore (amoore@asu.edu)

Supporting Information

Table of contents

1. Experimental section S3

1.1. General considerations and methods S3

1.2. Structural characterization S5

1.2.1. Compound **2**.. S5

1.2.2. Compounds **3-4** and synthetic intermediates S5

2. Nuclear magnetic resonance data S12

2.1. NMR spectroscopy of compound **2** S12

2.2. NMR spectroscopy of compound **3-4** and synthetic intermediates S13

3. Electrochemistry S34

3.1. Methods: cyclic voltammetry S34

3.2. Cyclic voltammetry of **1** and **3** S35

3.3. Effect of ethanol as weak hydrogen-bonding agent. Mathematical treatment. S36

4. Spectroelectrochemistry (IRSEC) data S39

5. Infrared (IR) data S40

6. Computational methods S41

7. References.. S50

1. Experimental section

1.1. General considerations and methods

Solvents like dichloromethane (DCM) and tetrahydrofuran (THF) were distilled prior to use; DCM was distilled from CaH_2 and THF from $LiAlH_4$. Other solvents like acetone, methanol (MeOH), diethyl ether (Et_2O) and acetonitrile (MeCN) were obtained from commercial sources and stored over molecular sieves (3 Å or 4 Å). All chemicals were purchased from Aldrich, Acros Organics or Alfa Aesar. Thin layer chromatography (TLC) was performed using silica-gel-coated glass plates from Merck Millipore. All column chromatography purifications were conducted with silica gel 60 (230–400 mesh). Solvents for column chromatography such as *n*-hexanes, DCM, ethyl acetate (EtOAc) and MeOH were distilled prior to use.

Mass spectra were obtained using a Voyager DE STR matrix-assisted laser desorption/ionization time-of-flight (MALDI-TOF) mass spectrometer. The spectra were taken in positive or negative ion reflector mode with *trans*, *trans*-1,4-diphenyl-1,3-butadiene as matrix. Positive or negative mode is specified in the observed ion mass.

High-resolution mass spectral analysis of the ester derivative (**3**) was performed using an Agilent 6530 Accurate-Mass Q-TOF LC-MS instrument. Electrospray ionization in positive mode was employed to generate the mass spectrum. The mobile phase consisted of methanol with 0.1% formic acid, delivered at a flow rate of 0.4 mL/min. A direct injection of 1 μL sample solution was employed, without using any chromatographic column. The mass spectrometer settings were optimized for the analysis, with a capillary voltage of 3500 V, gas temperature of 300 °C, drying gas flow rate of 8 L/min, nebulizer pressure of 35 psi, sheath gas temperature of 350 °C, sheath gas flow rate of 11 L/min, fragmentor voltage of 175 V, skimmer voltage of 60 V, and octupole RF voltage of 750 V. The mass range was scanned from 100 to 1000 m/z. Data acquisition was performed using Agilent MassHunter Data Acquisition software version B.08.00, and subsequent analysis was conducted with Agilent MassHunter Qualitative Analysis software version B.07.00.

Nuclear Magnetic Resonance (NMR) spectra were obtained with a 400 or 500 MHz Bruker spectrometer as it is indicated using standard pulse techniques. Deuterated solvents like chloroform ($CDCl_3$), acetone (Acetone-d_6), dimethyl sulfoxide (DMSO-d_6) and tetrahydrofuran (THF-d_8) were used as solvents for NMR characterization with TMS (0.05% v/v) as internal reference.

Structural characterization of **2-4**, and synthetic intermediates for **3** and **4** was done employing 1D (^{1}H and ^{13}C) NMR spectroscopy. Additionally, compound **4** was fully characterized employing 2D (COSY, HSQC and HMBC) NMR spectroscopy.

1.2. Structural characterization

1.2.1. Compound 2

Compound **2** (1,2,3,4-tetrahydro-1,4-methanoanthracene-9,10-dione)[1,2] was prepared following previously reported procedures. NMR data was identical to the cited reports. ^{1}H NMR (500 MHz, $CDCl_3$) δ 1.25 – 1.28 (m, 2H, H2/H3), 1.47 (dt, *J* = 9.1, 1.4 Hz, 1H, H11), 1.71 (dt, *J* = 9.0, 1.9, H11), 1.95 – 2.03 (m, 2H, H2/H3), 3.64 – 3.65 (m, 2H, H1 and H4), 7.66 – 7.70 (m, 2H, H6 and H7), 8.01 – 8.08 (m, 2H, H5 and H8).

1.2.2. Compounds 3-4 and synthetic intermediates

Reference compound methyl 9,10-dioxo-3,4,9,10-tetrahydro-1,4-methanoanthracene-1(2*H*)-carboxylate (**3**) and desired compound **4** (9,10-dioxo-3,4,9,10-tetrahydro-1,4-methanoanthracene-1(2*H*)-carboxylic acid) were synthetized following the synthetic steps presented in Scheme S1. In this strategy, the key Diels-Alder reaction[3,4] was performed between 1-methoxycarbonylcyclopenta-1,3-diene and 1,4-naphthoquinone (**1**) as dienophile. The diene was prepared *in-situ* from commercially available sodium cyclopentadienyl and methyl chloroformate (ClC(O)OMe) in THF under -78 °C.[5] Crude diene was used without purification in the Diels-Alder reaction with **1**, which after 5 days at room temperature (rt) was able to react and achieve the corresponding adduct **B** in 14% yield (considering both reaction steps). Diketone **B** was directly transformed into the dimethoxy-naphthalene derivative **C** with an excellent yield (90 %) employing KOH and MeI following a similar procedure previously described.[6,7]

Afterwards, intermediate **C** was hydrogenated employing standard conditions (H_2 with 20 mol% of $Pd(OH)_2$ in anhydrous MeOH) to achieve compound **D** (83% yield). This last intermediate was hydrolyzed under basic media into its corresponding carboxylic acid **E** (81% yield). A final well-known oxidation employing CAN (Cerium ammonium nitrate)[6] was successfully employed to achieve the desired final product (89% yield).

Additionally, a final esterification reaction, with EDC[8,9] as an activating reagent, was employed to obtain reference compound **3** (31 % yield).

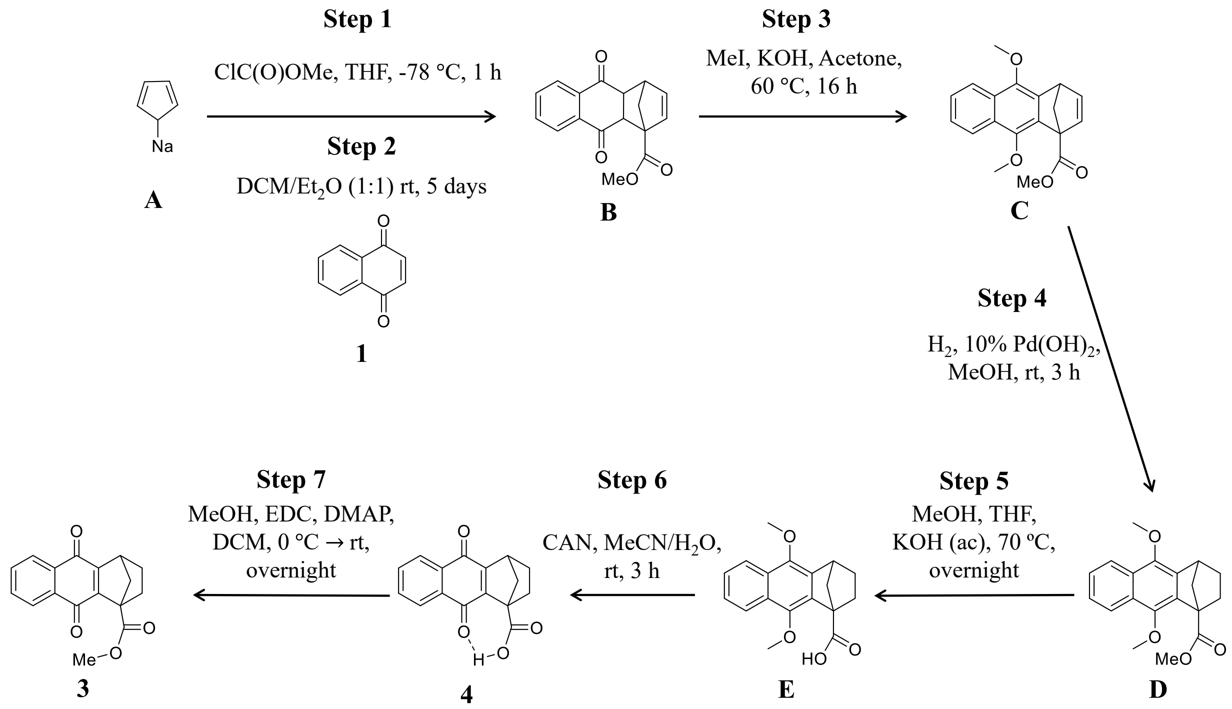

Scheme S1. Synthetic pathway to obtain compounds **3** and **4**.

1.2.2.1 Procedure and characterization of compounds 3-4 and synthetic intermediates

Step 1 - 2: Synthesis of diene and Diels-Alder reaction

Methyl 9,10-dioxo-4a,9,9a,10-tetrahydro-1,4-methanoanthracene-1(4*H*)-carboxylate (**B**). The title compound was synthesized in a two-step sequence with preparation of the diene first[5] and Diels-Alder second. In a three-necked round bottom flask, previously dried and equipped with a stirring bar, a commercially available solution of sodium cyclopentadienyl (11.9 mL of a solution 2.1 M in THF) was added together with freshly distilled THF (100 mL). After cooling down the solution until -78 °C under argon, 1.93 mL (25 mmol) of methyl chloroformate was added at such a rate that the reaction mixture always remained below -50 °C. After complete addition (around 10 min), the solution was allowed to slowly warm up to rt and then, stirring was further kept for an additional 1 h. The red solution was poured into water (500 mL) and the layers were carefully separated. Furthermore, the aqueous layer was subsequently washed with Et_2O (5 × 100 mL). Afterward, the combined organic layers were washed with water (5 × 100 mL) and saturated brine, and dried with $MgSO_4$ at 0 °C for 0.5 h. The final reaction crude was obtained after removal of the volatile fraction employing a rotary evaporator to give a dark yellow oil used in the next step without further purification.

This Diels-Alder adduct was made by dissolving 2.45 g (25 mmol) of 1,4-naphthoquinone (**1**) in anhydrous Et_2O and adding the crude methyl cyclopenta-1,3-diene-1-carboxylate previously obtained in DCM (ratio Et_2O: DCM – 1:1). This mixture was stirred for 5 days at rt under argon atmosphere. The resulting mixture was filtered through a pad of silica-gel, washed with copious amount of EtOAc (around 500 mL) and then the solvent was evaporated under reduced pressure. The resulting residue was purified twice by column chromatography on silica gel eluting with gradient of *n*-hexanes/EtOAc (90:10 → 70:30). The compound was isolated as a brown oil in 14% yield (0.988 g, 3.5 mmol), calculated from the two synthetic steps. ^1H NMR (500 MHz, $CDCl_3$) δ 1.82 (dd, *J* = 8.5, 1.8 Hz, 1H, H11/H11′), 1.91 – 1.95 (m, 1H, H11/H11′), 3.69 (dd, *J* = 9.3, 4.0 Hz, 1H, H4a), 3.74 – 3.76 (m, 1H, H4), 3.91 (s, 3H, OMe), 3.99 (d, *J* = 9.3 Hz, 1H, H9a), 6.07 (dd, *J* = 5.7, 2.9 Hz, 1H, H3), 6.26 (d, *J* = 5.7 Hz, 1H, H2), 7.67 – 7.76 (m, 2H, H6 and H7), 7.93 – 8.08 (m, 2H, H5 and H8). MS (MALDI-TOF): m/z 282.085 (calcd. for $C_{17}H_{14}O_4^+$, M^+, 282.089).

Step 3: Methylation reaction

Methyl 9,10-dimethoxy-1,4-methanoanthracene-1(4H)-carboxylate (**C**). The title compound was synthesized following procedures previously reported with some modifications.[6,7,10] A solution of diketone **C** (0.890 g, 3.15 nmol) in dry acetone (100 mL) was stirred and refluxed under argon atmosphere. To the previously mentioned solution, methyl iodide (2.76 mL) was added over a five-minute period. Then, a 10% methanolic KOH solution was added dropwise by syringe (each drop was followed by a transient purple coloration), until no more purple discharges could be seen. The inert atmosphere was continuously maintained through the entire reaction time (16 h in total). The pale-yellow suspension obtained was dissolved in 2M aqueous HCl (20 mL) and extracted with DCM (4 × 50 mL). The organic layers were combined and dried over Na_2SO_4; the solvent was removed by evaporation at reduced pressure to give a yellow oil. Desired compound **C** was purified by column chromatography on silica gel eluting with gradient of *n*-hexanes/EtOAc (90:10 → 80:20). The compound was isolated as a yellow solid in 90% yield (0.880 g, 2.84 mmol). ^{1}H NMR (500 MHz, $CDCl_3$) δ 2.48 (dd, *J* = 7.6, 1.7 Hz, 1H, H11/H11′), 2.59 – 2.64 (m, 1H, H11/H11′), 3.80 (s, 3H, 10-OMe), 3.93 (s, 3H, OMe, ester), 3.99 (s, 3H, 9-OMe), 4.37 – 4.38 (m, 1H, H4), 6.79 (dd, *J* = 5.4, 3.2 Hz, 1H, H3), 7.01 (d, *J* = 5.4 Hz, 1H, H2), 7.44 - 7.48 (m, 2H, H6 and H7), 7.90 – 7.99 (m, 1H, H5), 7.99 – 8.09 (m, 1H, H8). ^{13}C NMR (126 MHz, $CDCl_3$) δ 46.90 (C4), 52.24 (OMe ester), 62.11 (OMe-C9), 62.58 (C1), 63.22 (OMe-C10), 67.85 (CH_2, C11), 122.05 (C5), 122.36 (C8), 125.87 (C6/C7), 125.87 (C6/C7), 127.97 (C10a), 128.42 (C8a), 134.28 (C9a), 136.76 (C4a), 140.39 (C2), 141.87 (C3), 145.20 (C10), 146.01 (C9), 172.38 (C(O) ester). MS (MALDI-TOF): m/z 310.120 (calcd. for $C_{19}H_{18}O_4^+$, M^+, 310.120).

Step 4: Hydrogenation reaction

Methyl 9,10-dimethoxy-3,4-dihydro-1,4-methanoanthracene-1(2*H*)-carboxylate (D). The title compound was synthesized following a previously reported procedure.[6] A solution of the previously isolated compound **C** (0.812 g, 2.62 mmol) in anhydrous MeOH (180 mL) was prepared and thoroughly degassed with nitrogen, afterwards 10% of palladium on carbon (80 mg) was added in small portions. The mixture was then hydrogenated at 40 psi for 3 h. Analysis by TLC indicated full conversion of the starting material after that period. The reaction mixture was then carefully filtered through

celite and washed with MeOH (500 mL). Afterwards, the solvent was evaporated under reduced pressure, and the resulting residue was purified by column chromatography on silica gel eluting with DCM to give a light-yellow solid of desired compound **D** in 83% yield (0.679 g, 2.17 mmol). ^{1}H NMR (500 MHz, $CDCl_3$) δ 1.46 - 1.52 (m, 1H, H3/H3´), 1.67 - 1.72 (m, 1H, H2/H2´), 1.93 (dd, *J* = 9.0, 1.4 Hz, 1H, H11/H11´), 2.19 (tt, *J* = 11.4, 4.2, Hz, 1H, H3/H3´), 2.22 – 2.25 (m, 1H, H11/H11´), 2.37 (ddd, *J* = 12.2, 11.2, 4.2 Hz, 1H, H2/H2´), 3.84 (s, 4H, 10-OMe + H4), 3.88 (s, 3H, OMe ester), 4.00 (s, 3H, 9-OMe), 7.45 – 7.48 (m, 2H, H6 + H7), 7.98 – 8.07 (m, 1H, H5), 8.08 – 8.17 (m, 1H, H8). ^{13}C NMR (126 MHz, $CDCl_3$) δ 28.82 (C2), 29.88 (C3), 40.70 (C4), 51.56 (C11), 52.08 (OMe ester), 56.69 (C1), 61.84 (OMe C9), 62.62 (OMe C10), 122.11 (C5), 122.33 (C8), 125.39 (C6/C7), 125.43 (C6/C7), 128.25 (C10a), 128.71 (C8a), 134.04 (C4a), 135.98 (C9a), 144.05 (C10), 144.86 (C9), 174.35 (C(O) ester). MS (MALDI-TOF): m/z 312.132 (calcd. for $C_{19}H_{20}O_4^+$, M^+, 312.136).

Step 5: Ester hydrolysis

9,10-Dimethoxy-3,4-dihydro-1,4-methanoanthracene-1(2*H*)-carboxylic acid (**E**). The title compound was synthesized following previously reported methodology.[6] A mixture of the ester **D** (500 mg, 1.6 mmol), MeOH (13 mL), 10% aqueous KOH (9 mL) and freshly distilled THF (27 mL) was stirred at rt overnight. Analysis by TLC showed remaining starting material, therefore additional amount of KOH was added (18 mL of 10% aqueous solution in total) and the solution was heated at 70 °C overnight. Full conversion of the starting material was achieved after this procedure. The reaction mixture was quenched with a 2M aqueous HCl solution (100 mL), extracted with EtOAc (3 × 100 mL), dried over Na_2SO_4 and concentrated by evaporation under reduced pressure to give the crude reaction. Pure acid **E** (81% yield, 0.387 g, 1.3 mmol) was obtained after purification by column chromatography on silica gel eluting with gradient of *n*-hexanes/EtOAc (90:10 → 50:50). ^{1}H NMR (500 MHz, Acetone-d_6) δ 1.43 – 1.48 (m, 1H, H3/H3´), 1.66 – 1.65 (m, 1H, H2/H2´), 1.98 (dd, *J* = 8.9, 1.5 Hz, 1H, H11/H11´), 2.15 (dq, *J* = 8.8, 2.0 Hz, 1H, H11/H11´), 2.19 – 2.25 (m, 1H, H3/H3´), 2.35 (td, *J* = 11.9, 4.2 Hz, 1H, H2/H2´), 3.82 (s, 3H, 10-OMe), 3.84 – 3.88 (m, 1H, H4), 4.00 (s, 3H, 9-OMe), 7.44 – 7.50 (m, 2H, H6 + H7), 7.99 – 8.04 (m, 1H, H5), 8.08 - 8.11 (m, 1H, H8), 10.94 (br. s, 1H, OH of carboxylic acid). ^{13}C NMR (126 MHz, Acetone-d_6) δ 28.68 (C2, overlap

with the residual signal of the solvent), 29.75 (C3, overlap with the residual signal of the solvent), 40.63 (C4), 51.41(C11), 56.65 (C1), 61.07 (OMe C9), 61.89 (OMe C10), 121.93 (C5), 122.21 (C8), 125.24 (C6 + C7), 128.38 (C10a), 128.68 (C8a), 134.22 (C4a), 136.19 (C9a), 144.09 (C10), 144.72 (C9), 173.98 (C(O) carboxylic acid). MS (MALDI-TOF): m/z 298.119 (calcd. for $C_{18}H_{18}O_4^+$, M^+, 298.120).

Step 6: Oxidation

9,10-Dioxo-3,4,9,10-tetrahydro-1,4-methanoanthracene-1(2*H*)-carboxylic acid (**4**). The title compound was synthesized employing reported methodology.[6] A solution of ceric ammonium nitrate (CAN, 2.383 g, 4.35 mmol) in MeCN:H_2O (1:1 ratio, 2 mL) was added to the carboxylic acid precursor **E** (273.3 mg, 0.725 mmol) dissolved in 10 mL of MeCN. The solution was kept under argon and the CAN solution was added dropwise in a period of 10 minutes. The reaction was allowed to proceed for 3 h with stirring under argon. The progress of the reaction was monitored by TLC and by the disappearance of the blue fluorescence which is characteristic of the starting material. After 3 h, total conversion of the starting material was observed, and the workup was done by extraction with EtOAc (3 × 50 mL)/H_2O. Afterwards, the organic phases were combined, dried over Na_2SO_4 and concentrated by evaporation under reduced pressure to give the crude reaction product. Purification by flash column chromatography on silica gel eluting with DCM/MeOH (98:2) yielded the pure compound **3** (173.2 mg, 89%) as a yellow powder. ^1H NMR (400 MHz, DMSO-d_6) δ 1.25 – 1.32 (m, 1H, H3/H3′), 1.36 – 1.43 (m, 1H, H2/H2′), 1.75 (dd, J = 8.8, 1.3 Hz, 1H, H11/H11′), 1.93 (dd, J = 8.8, 2.2 Hz, 1H, H11/H11′), 2.03 – 2.12 (m, 1H, H3/H3′), 2.19 (ddd, J = 11.5, 9.3, 3.7 Hz, 1H, H2/H2′), 3.49 - 3.50 (m, 1H, H4), 7.77 – 7.87 (m, 2H, H6 + H7), 7.89 – 7.99 (m, 2H, , H5 + H8), 12.66 (s, 1H, OH acid). ^1H NMR (400 MHz, THF-d_8) δ 1.31 – 1.37 (m, 1H, H3/H3′), 1.43 – 1.50 (m, 1H, H2/H2′), 1.74 – 1.78 (m, 1H, H11/H11′, overlap with residual signal of THF-d_8), 2.01 – 2.05 (m, 1H, H11/H11′), 2.09 – 2.16 (m, 1H, H3/H3′), 2.29 (ddd, J = 12.1, 9.5, 4.0 Hz, 1H, H2/H2′), 3.55 – 3.56 (m, 1H, H4, overlap with residual signal of THF-d_8), 7.69 - 7.73 (m, 2H, H6 + H7), 7.96 – 8.03 (m, 2H, H5 + H8), 11.19 (br s, 1H, OH acid). ^{13}C NMR (101 MHz, THF-d_8) δ 27.59 (C3), 27.75 (C2), 41.73 (C4), 52.11 (C11), 59.14 (C1), 126.32 (C5/C8), 126.46 (C5/C8), 133.70 (C6/C7), 133.80 (C6/C7), 133.95 (C8a/C10a), 134.12 (C8a/C10a), 152.43 (C9a), 154.52 (C4a), 172.37

(C(O) acid), 180.88 (10-C(O)), 181.72 (9-C(O)). MS (MALDI-TOF): m/z 268.071 (calcd. for $C_{16}H_{12}O_4^+$, M^+, 268.073).

Step 7: Esterification

Methyl 9,10-dioxo-3,4,9,10-tetrahydro-1,4-methanoanthracene-1(2*H*)-carboxylate (**3**). The desired reference compound was obtained following an esterification methodology previously used.[8] Compound **4** (134 mg, 0.5 mmol) and 1-(3-dimethylaminopropyl)-3-ethyl carbodiimide hydrochloride, EDC (85 mg, 0.55 mmol, 1.1 eq.) were dissolved in anhydrous DCM (1.5 mL) and stirred for 5 minutes at 0 °C. Subsequently, MeOH (130 µL, 4 mmol, 8 eq.) and DMAP (5 mg, 10% mol) were added to the reaction mixture and stirred for 15 minutes at 0 °C. The reaction was kept stirred overnight at rt. The solution was washed twice with 0.5M HCl, then saturated $NaHCO_3$ solution, and dried over $MgSO_4$. The organic solvent was removed under reduced pressure and chromatographed (silica, hexane/ethyl acetate, 9:1) to give **4** (44 mg) in 31 % yield. ^{1}H-NMR (500 MHz, $CDCl_3$) δ 1.36 – 1.40 (m, 1H, H3/H3´), 1.57 – 1.60 (m, 1H, H2/H2´, overlap with the residual peak of water from $CDCl_3$), 1.78 (d, J = 8.4 Hz, 1H, H11/H11´), 2.11 – 2.19 (m, 2H, H11/H11´ + H3/H3´), 2.38 (ddd, J = 12.8, 9.6, 4.0 Hz, 1H, H2/H2´), 3.68 – 3.69 (m, 1H, H4), 3.85 (s, 3H, OMe), 7.70 (dd, J = 5.6, 3.3 Hz, 2H, H6 + H7), 8.04 (ddd, J = 18.2, 5.6, 3.4 Hz, 2H, H5 + H8). ^{13}C NMR (126 MHz, $CDCl_3$) δ 27.10 (C3), 27.31(C2), 41.15 (C4), 51.77 (OMe ester), 52.44 (C11), 58.28 (C1), 126.42 (C5/C8), 126.48 (C5/C8), 133.02 (C6/C7), 133.08 (C6/C7), 133.58 (C8a/C10a), 133.73 (C8a/C10a), 151.93 (C9a), 154.11 (C4a), 172.11(C(O) ester), 180.88 (10-C(O)), 181.95 (9-C(O)). HRMS (MALDI-TOF): m/z 283.096 (calcd. for $C_{17}H_{15}O_4^+$, M^+, 283.097).

2. Nuclear magnetic resonance data

2.1. NMR spectroscopy of compound 2

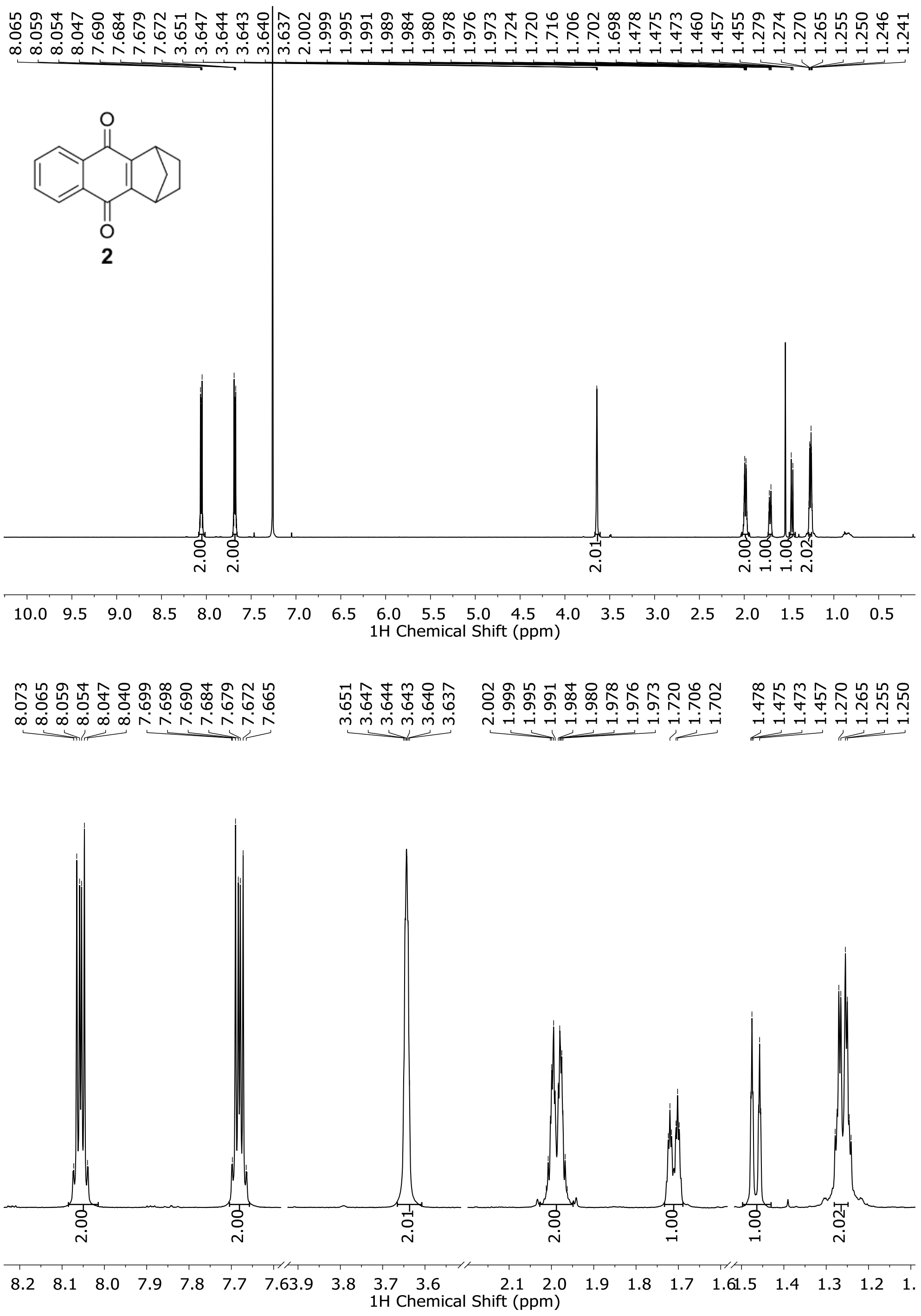

Figure S1. ¹H NMR (500 MHz, $CDCl_3$) full spectrum of 1,2,3,4-tetrahydro-1,4-methanoanthracene-9,10-dione (**2**) (top) and expansion of selected regions (bottom).

2.2. NMR spectroscopy of compound 3-4 and synthetic intermediates

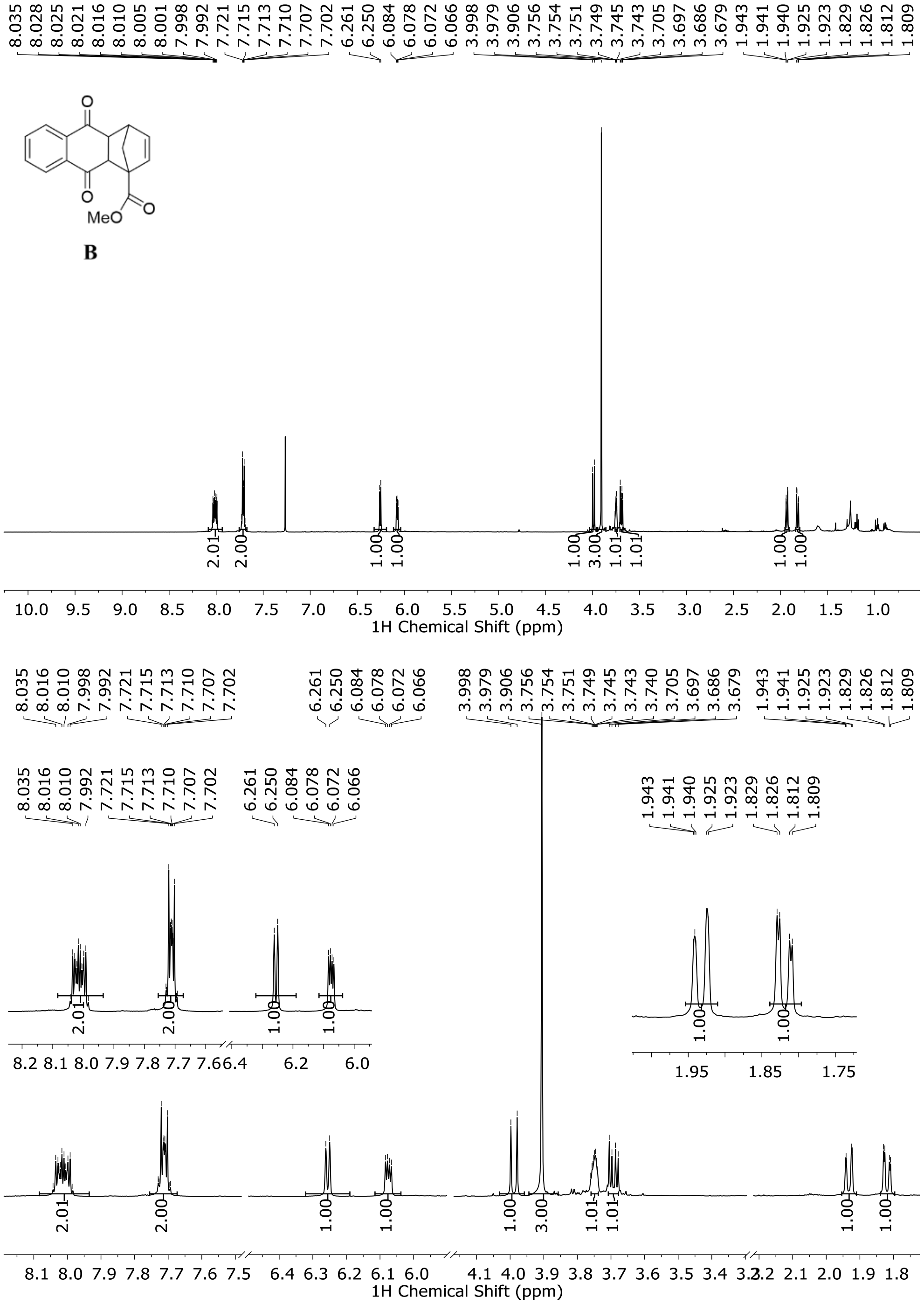

Figure S2. ^{1}H NMR (500 MHz, $CDCl_3$) full spectrum of methyl 9,10-dioxo-4a,9,9a,10-tetrahydro-1,4-methanoanthracene-1(4*H*)-carboxylate (**B**) (top) and expansion of selected regions (bottom, additional expansions are included in the inset).

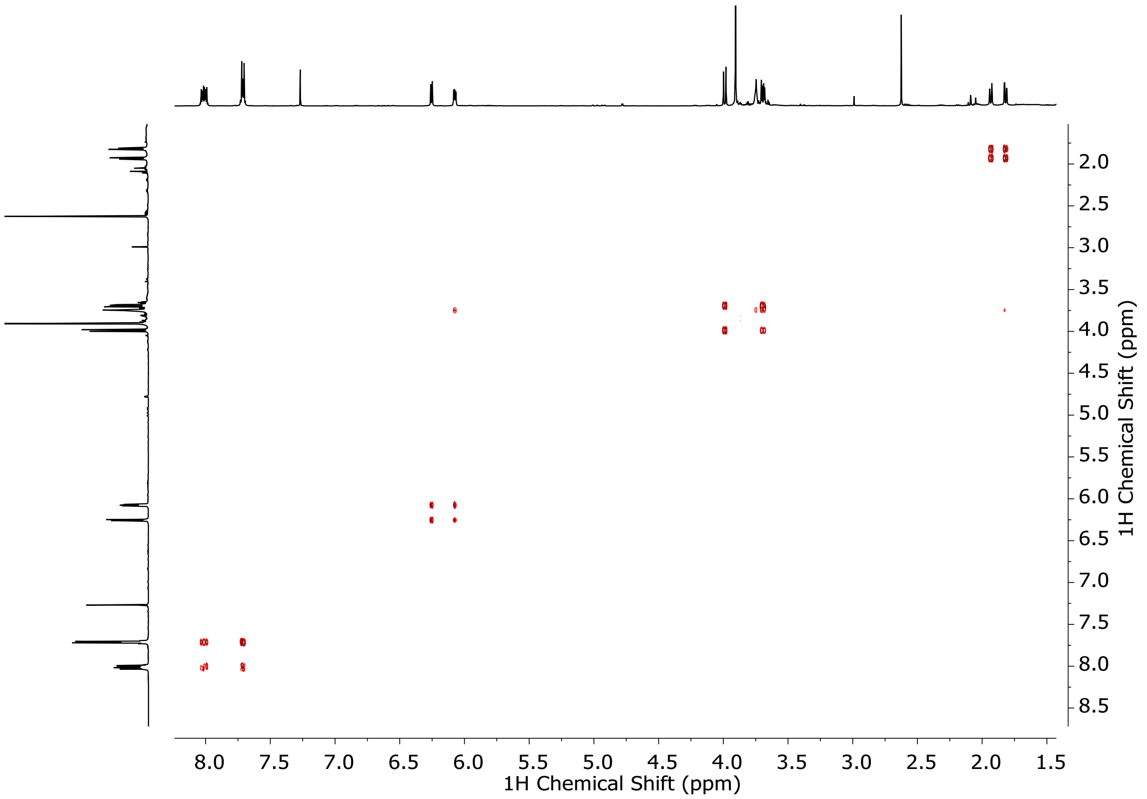

Figure S3. ^{1}H-^{1}H COSY (500 MHz, $CDCl_3$) full spectrum of methyl 9,10-dioxo-4a,9,9a,10-tetrahydro-1,4-methanoanthracene-1(4*H*)-carboxylate (**B**).

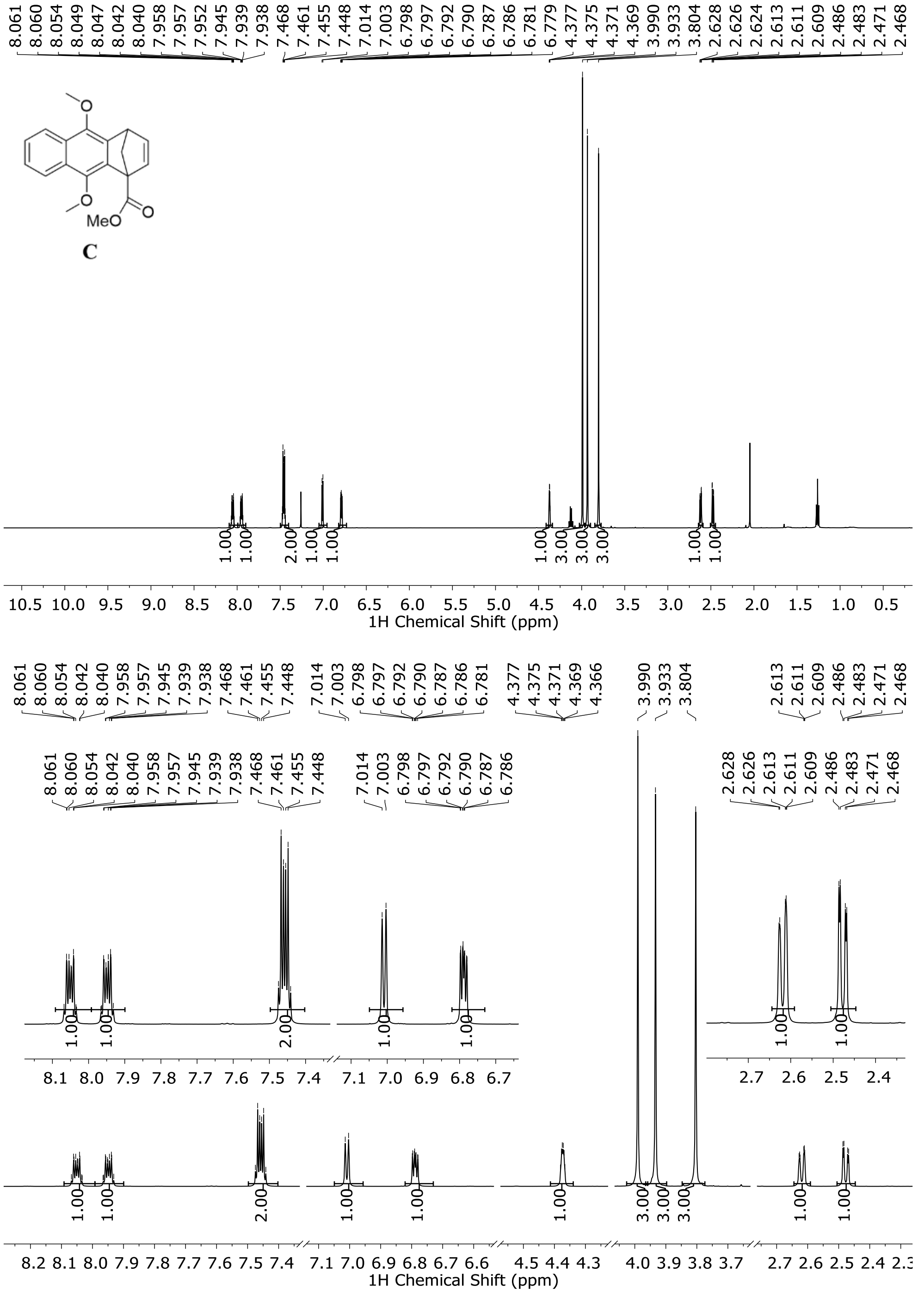

Figure S4. ^{1}H NMR (500 MHz, $CDCl_3$) full spectrum of methyl 9,10-dimethoxy-1,4-methanoanthracene-1(4*H*)-carboxylate (**C**) (top) and expansion of selected regions (bottom, additional expansions are included in the inset).

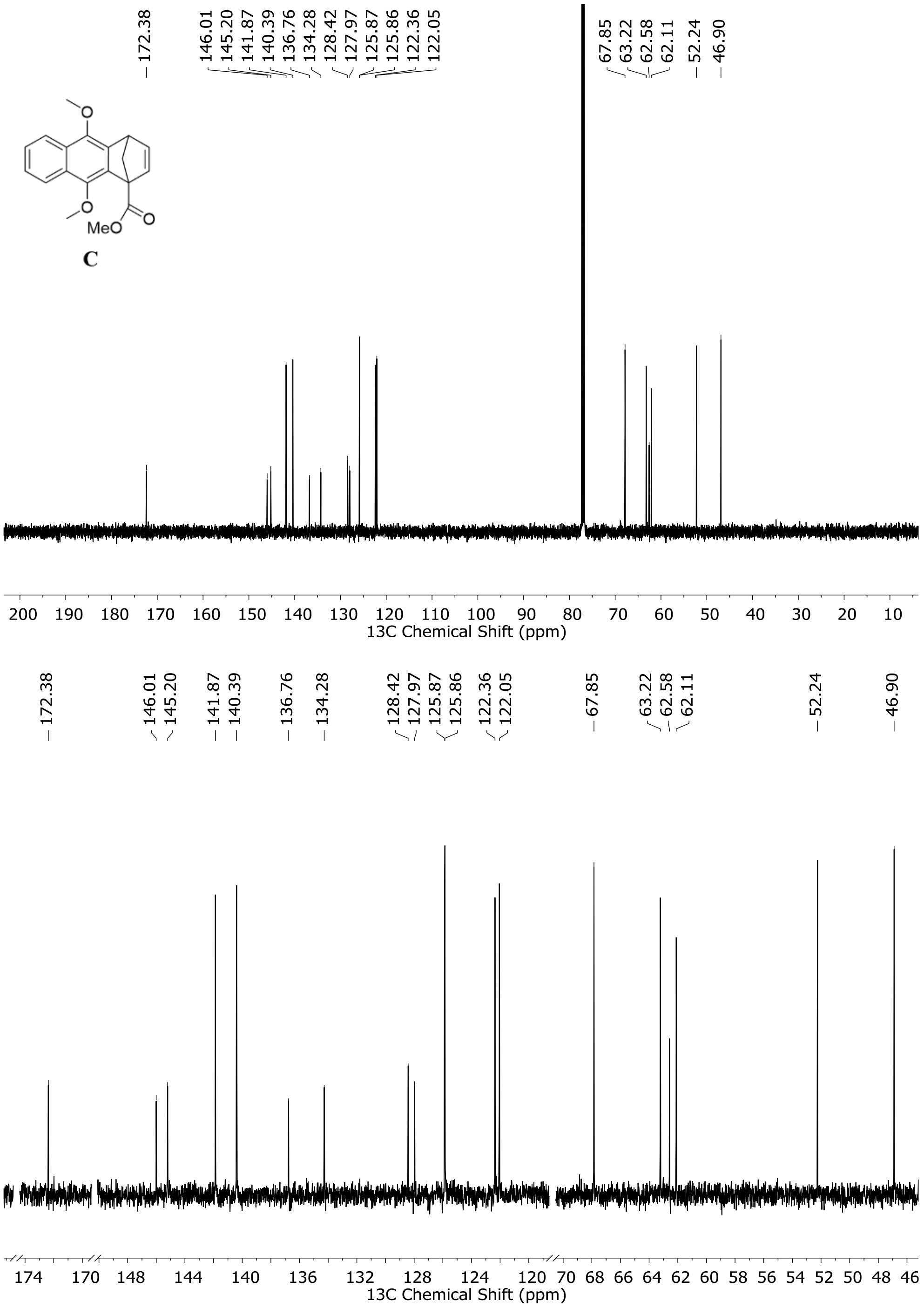

Figure S5. ^{13}C NMR (126 MHz, $CDCl_3$) full spectrum of methyl 9,10-dimethoxy-1,4-methanoanthracene-1(4*H*)-carboxylate (**C**) (top) and expansion of selected regions (bottom).

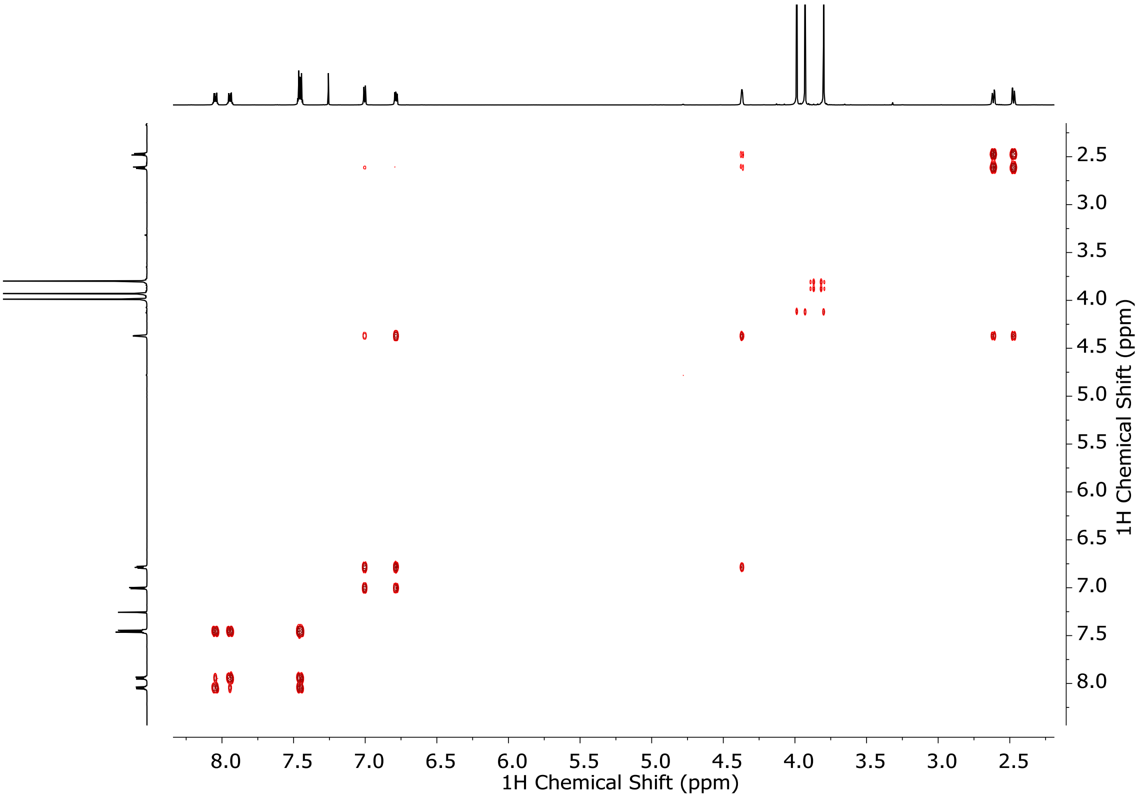

Figure S6. ^{1}H-^{1}H COSY (500 MHz, $CDCl_3$) spectrum of methyl 9,10-dimethoxy-1,4-methanoanthracene-1(4*H*)-carboxylate (**C**).

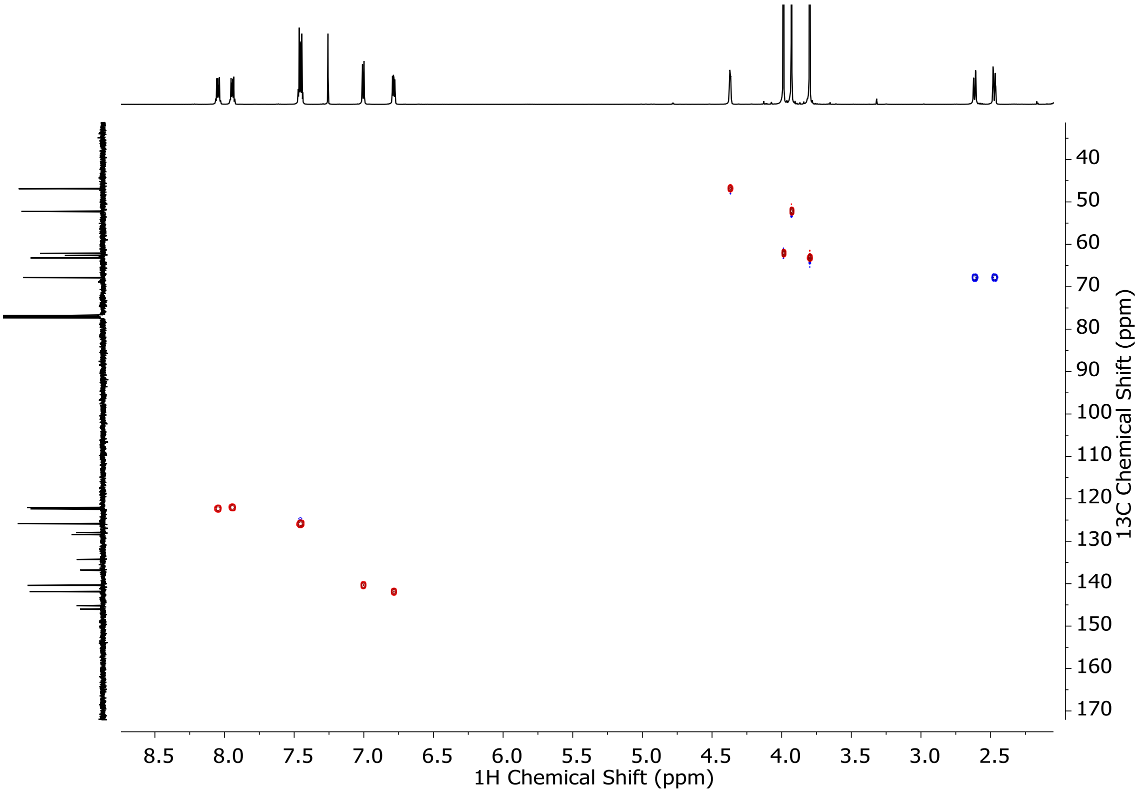

Figure S7. ^{1}H-^{13}C HSQC (500 MHz, $CDCl_3$) spectrum of methyl 9,10-dimethoxy-1,4-methanoanthracene-1(4*H*)-carboxylate (**C**).

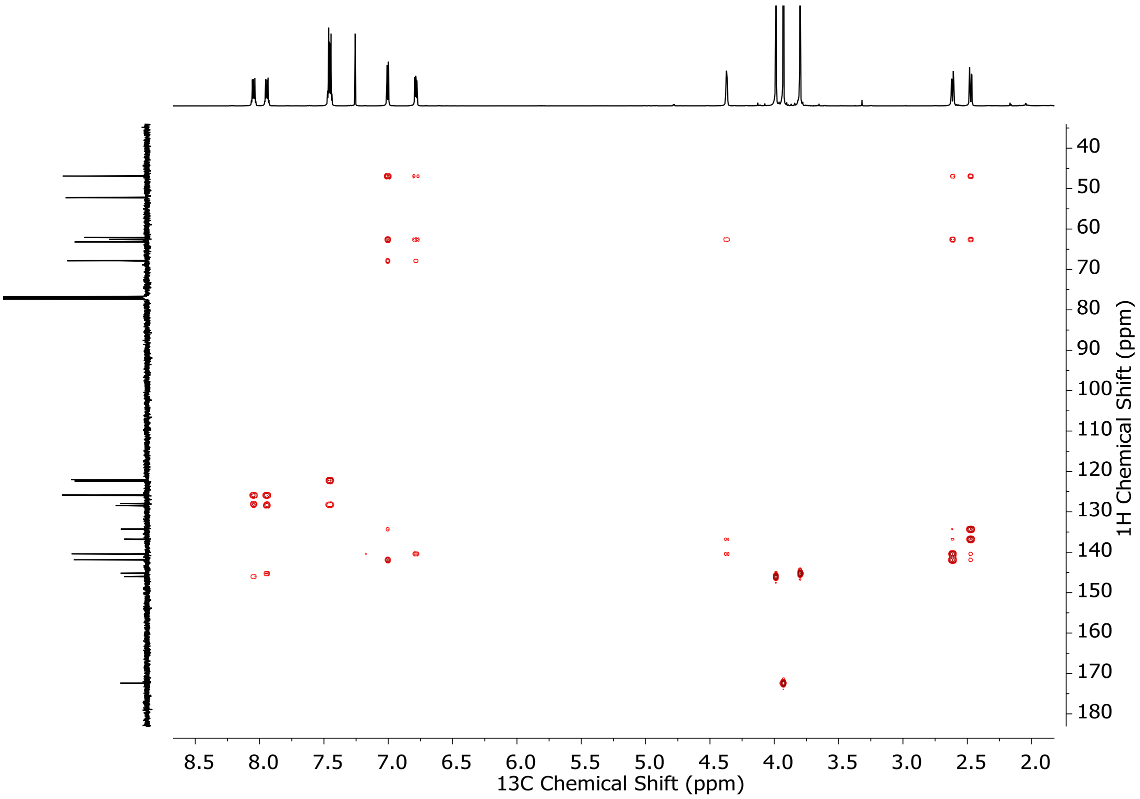

Figure S8. ^{1}H-^{13}C HMBC (500 MHz, $CDCl_3$) spectrum of methyl 9,10-dimethoxy-1,4-methanoanthracene-1(4*H*)-carboxylate (**C**).

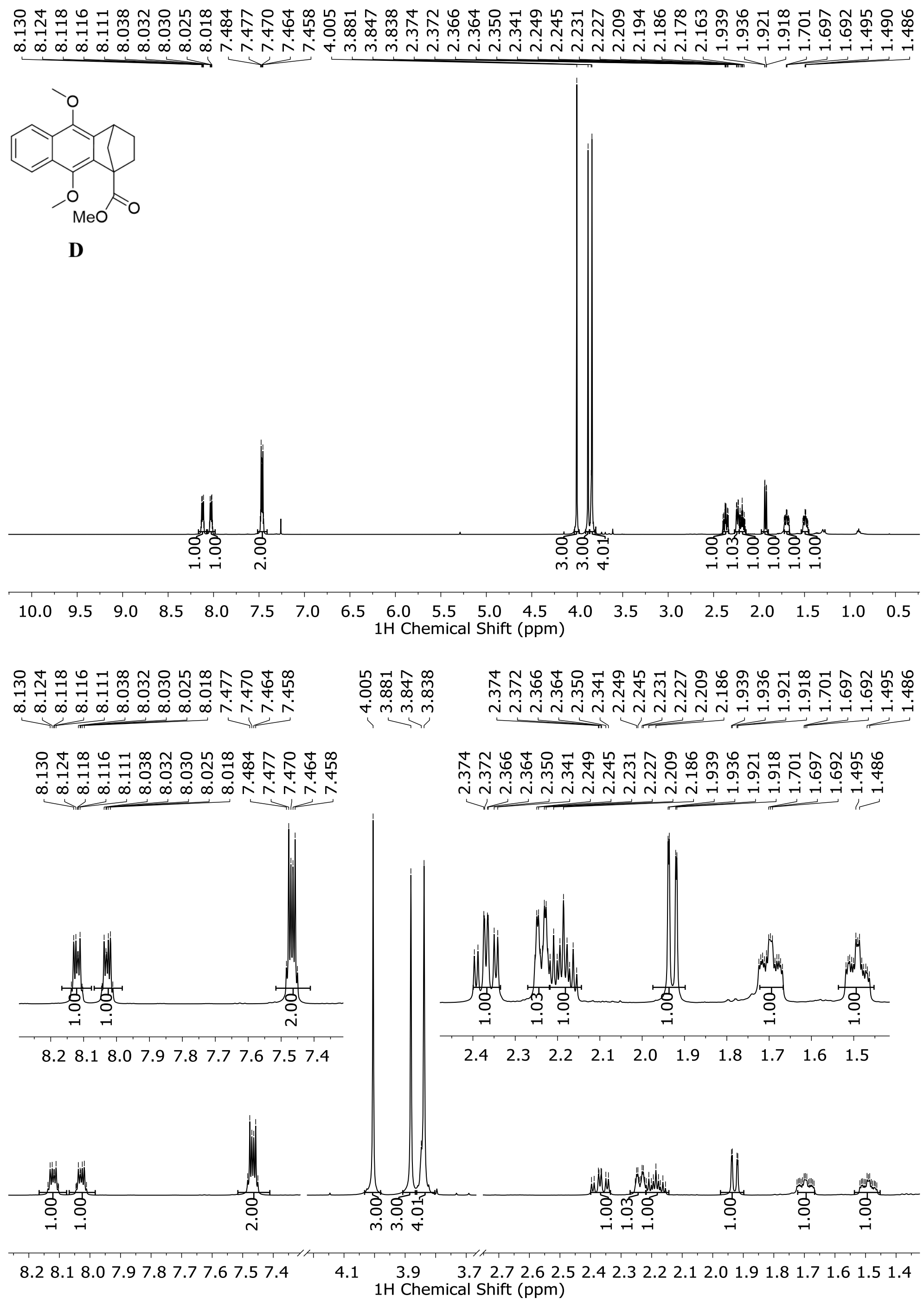

Figure S9. ^{1}H NMR (500 MHz, $CDCl_3$) full spectrum of methyl 9,10-dimethoxy-3,4-dihydro-1,4-methanoanthracene-1(2*H*)-carboxylate (**D**) (top) and expansion of selected regions (bottom, additional expansions are included in the inset).

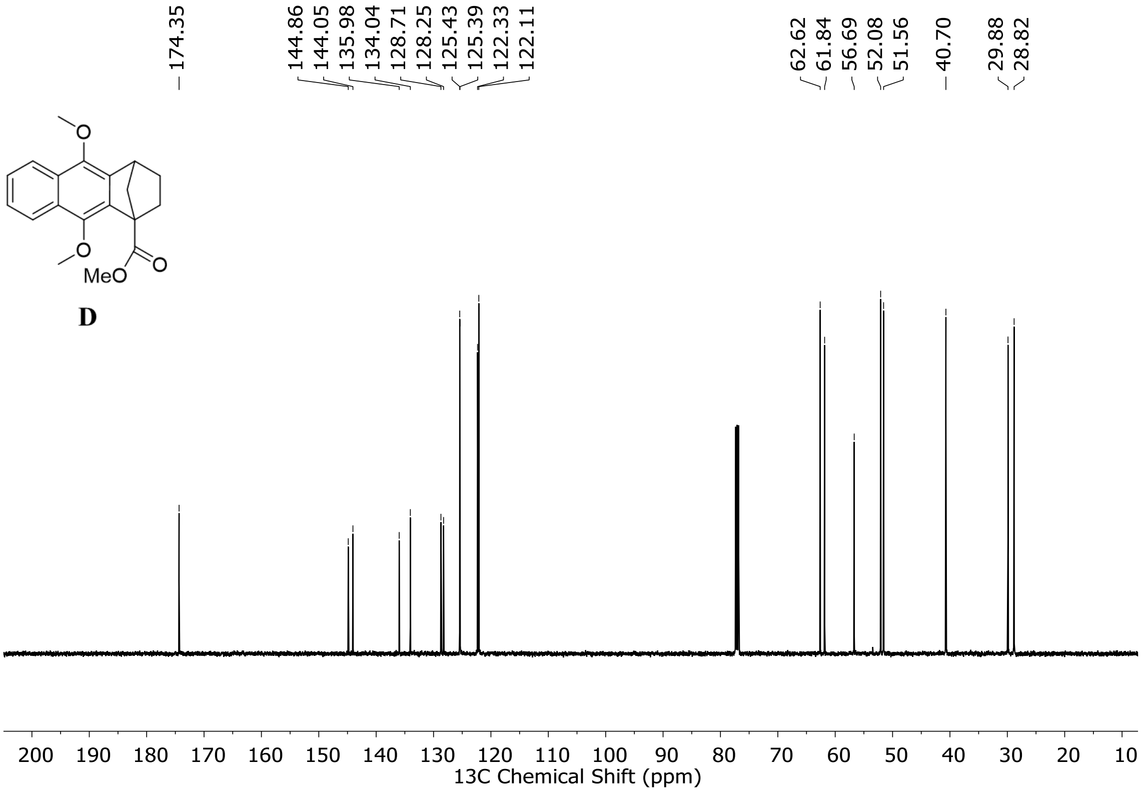

Figure S10. ^{13}C NMR (126 MHz, $CDCl_3$) full spectrum of methyl 9,10-dimethoxy-3,4-dihydro-1,4-methanoanthracene-1(2*H*)-carboxylate (**D**).

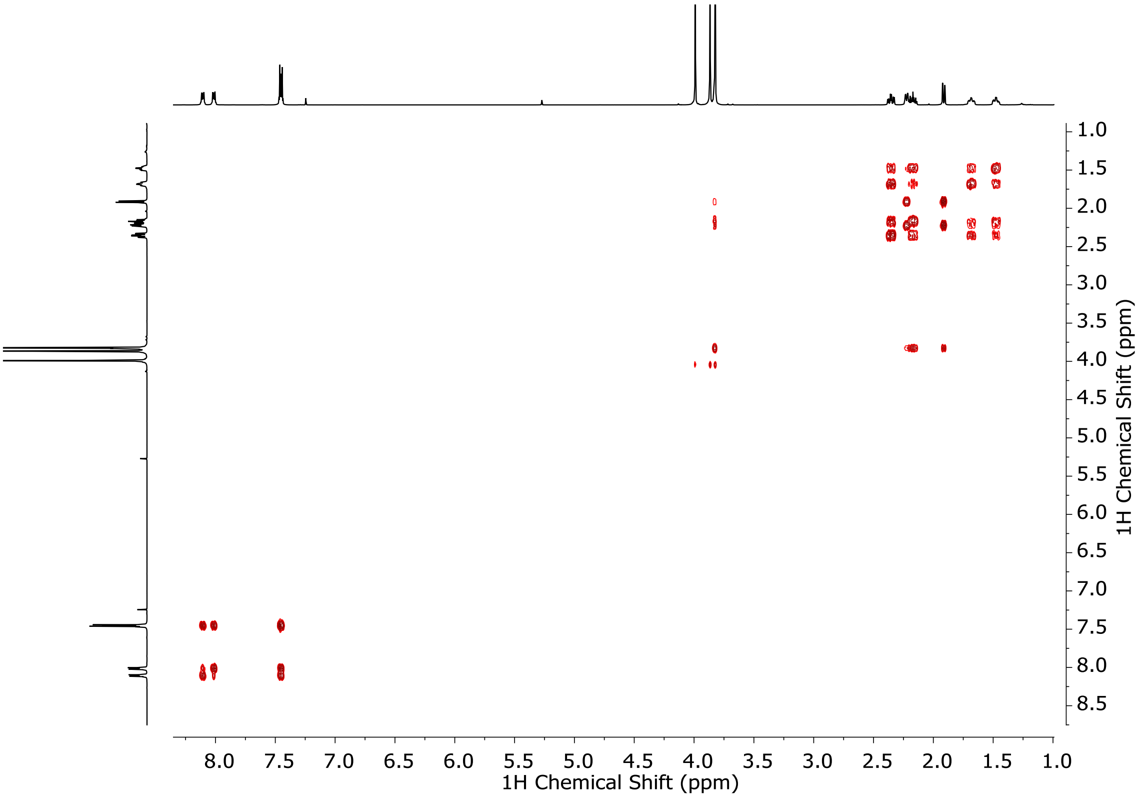

Figure S11. ^{1}H-^{1}H COSY (500 MHz, $CDCl_3$) spectrum of methyl 9,10-dimethoxy-3,4-dihydro-1,4-methanoanthracene-1(2*H*)-carboxylate (**D**).

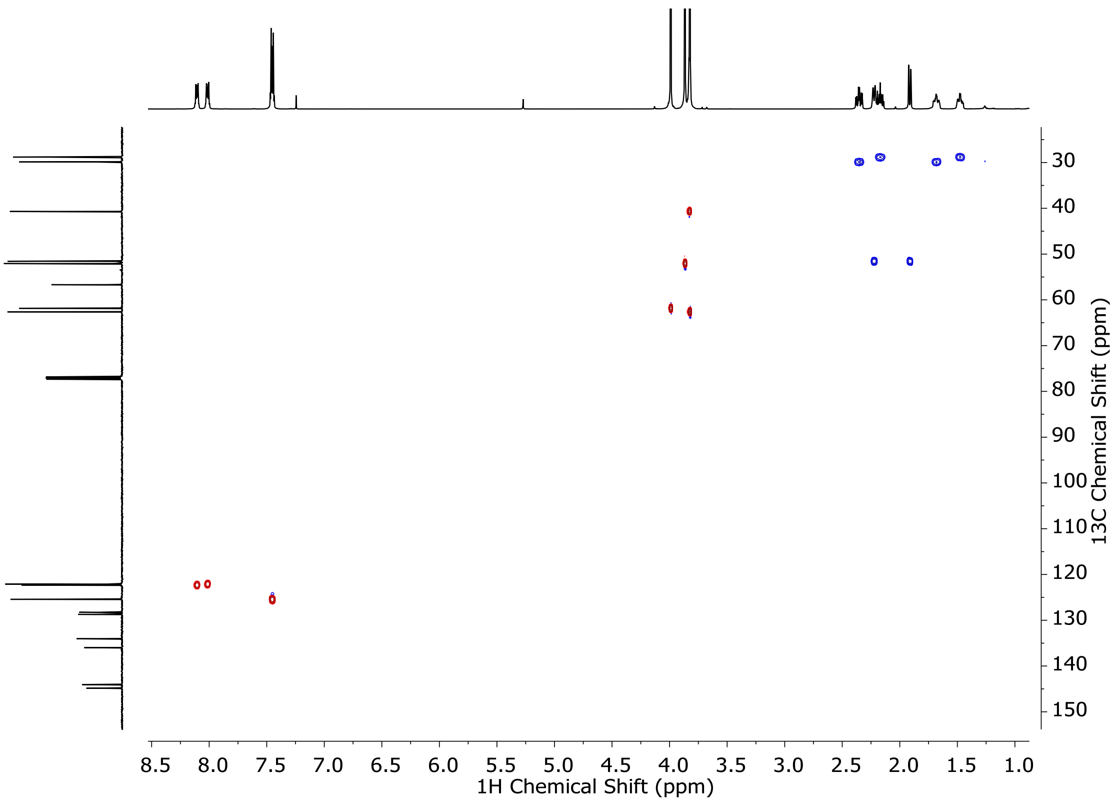

Figure S12. ^{1}H-^{13}C HSQC (500 MHz, $CDCl_3$) spectrum of methyl 9,10-dimethoxy-3,4-dihydro-1,4-methanoanthracene-1(2*H*)-carboxylate (**D**).

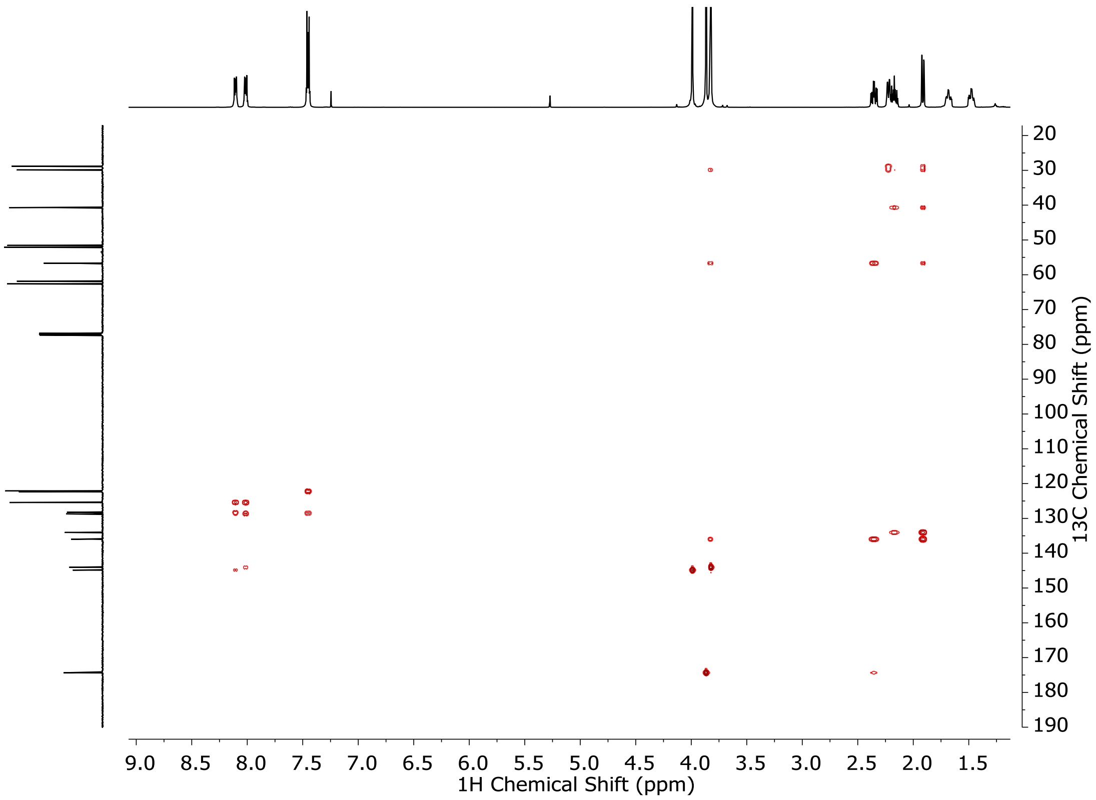

Figure S13. ^{1}H-^{13}C HMBC (500 MHz, $CDCl_3$) spectrum of methyl 9,10-dimethoxy-3,4-dihydro-1,4-methanoanthracene-1(2*H*)-carboxylate (**D**).

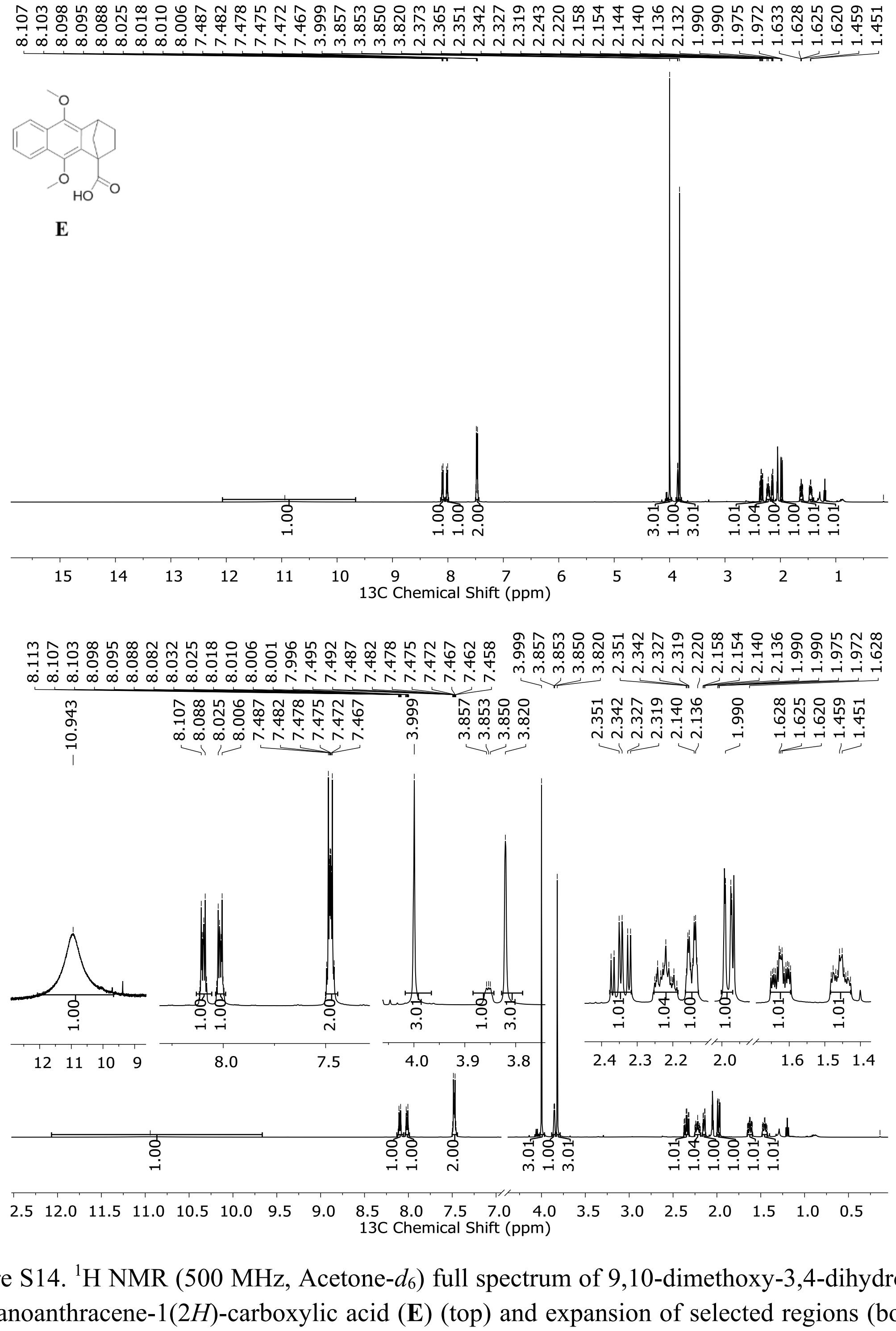

Figure S14. ^{1}H NMR (500 MHz, Acetone-d_6) full spectrum of 9,10-dimethoxy-3,4-dihydro-1,4-methanoanthracene-1(2*H*)-carboxylic acid (**E**) (top) and expansion of selected regions (bottom, additional expansions are included in the inset).

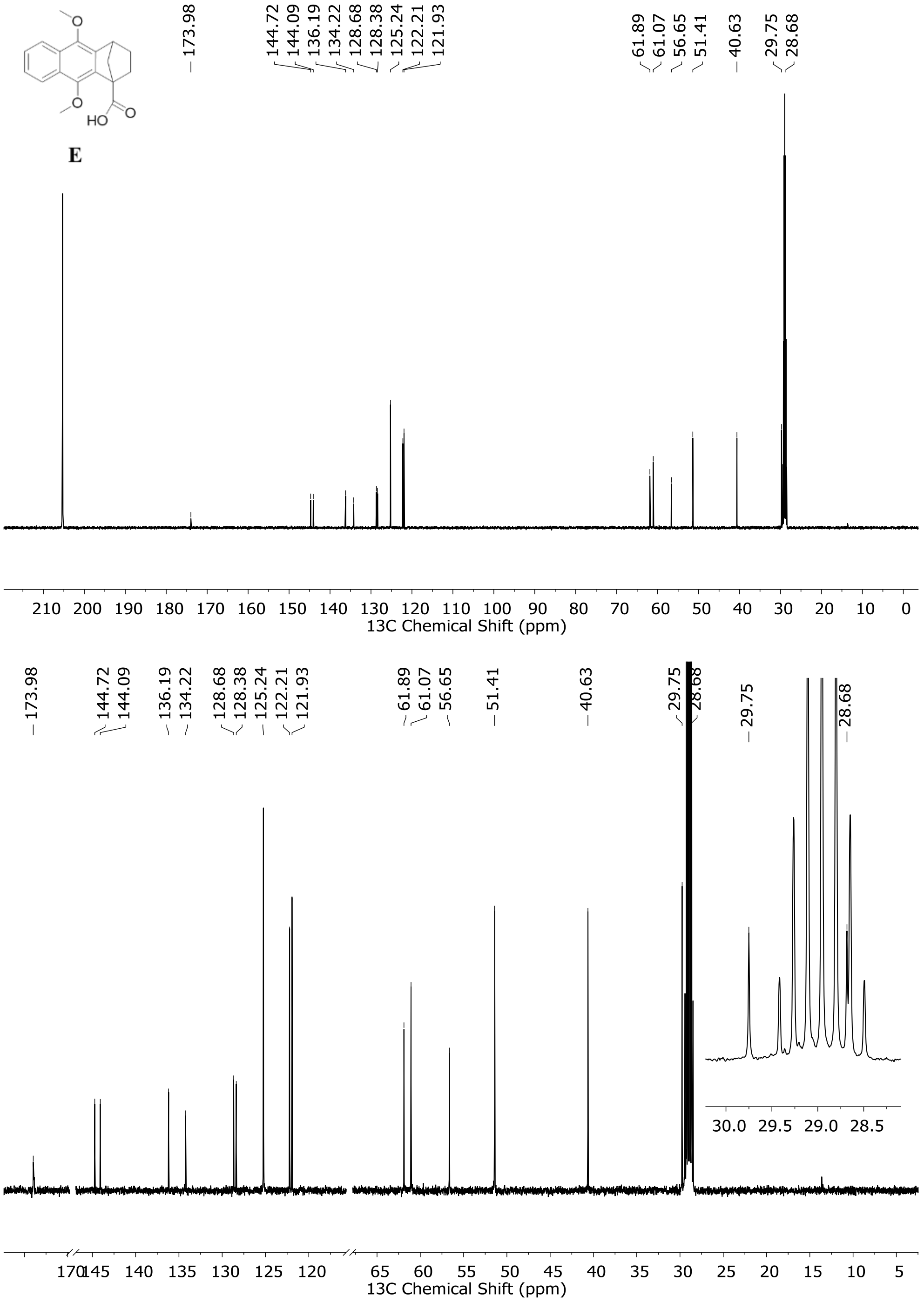

Figure S15. ^{13}C NMR (126 MHz, Acetone-d_6) full spectrum of 9,10-dimethoxy-3,4-dihydro-1,4-methanoanthracene-1(2*H*)-carboxylic acid (**E**) (top) and expansion of selected regions (bottom, additional expansions are included in the inset).

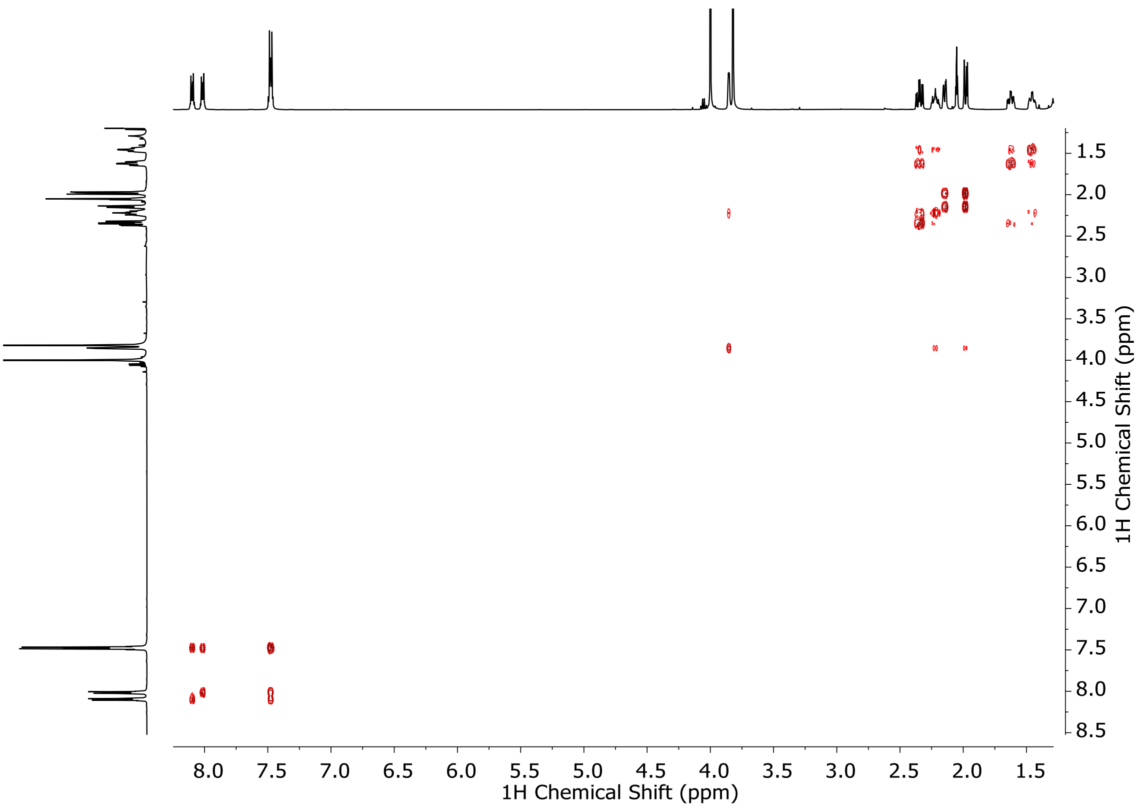

Figure S16. ^{1}H-^{1}H COSY (500 MHz, Acetone-d_6) spectrum of 9,10-dimethoxy-3,4-dihydro-1,4-methanoanthracene-1(2*H*)-carboxylic acid (**E**).

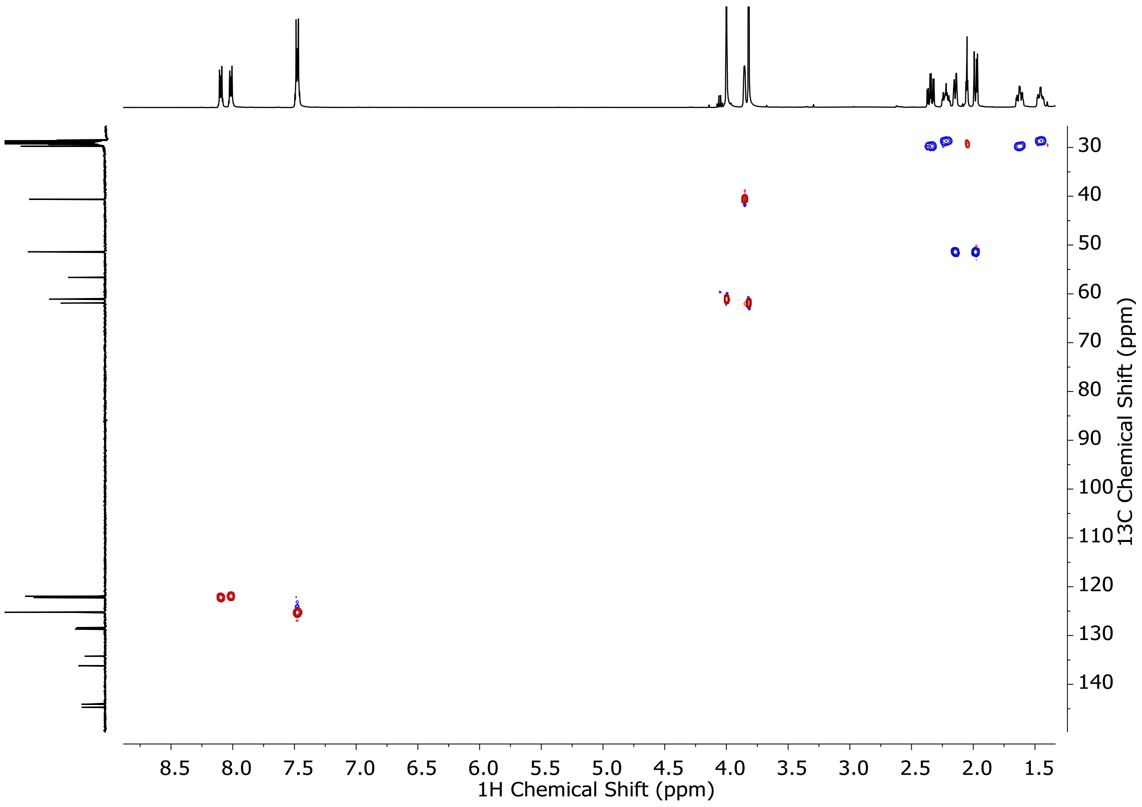

Figure S17. ^{1}H-^{13}C HSQC (500 MHz, Acetone-d_6) spectrum of 9,10-dimethoxy-3,4-dihydro-1,4-methanoanthracene-1(2*H*)-carboxylic acid (**E**).

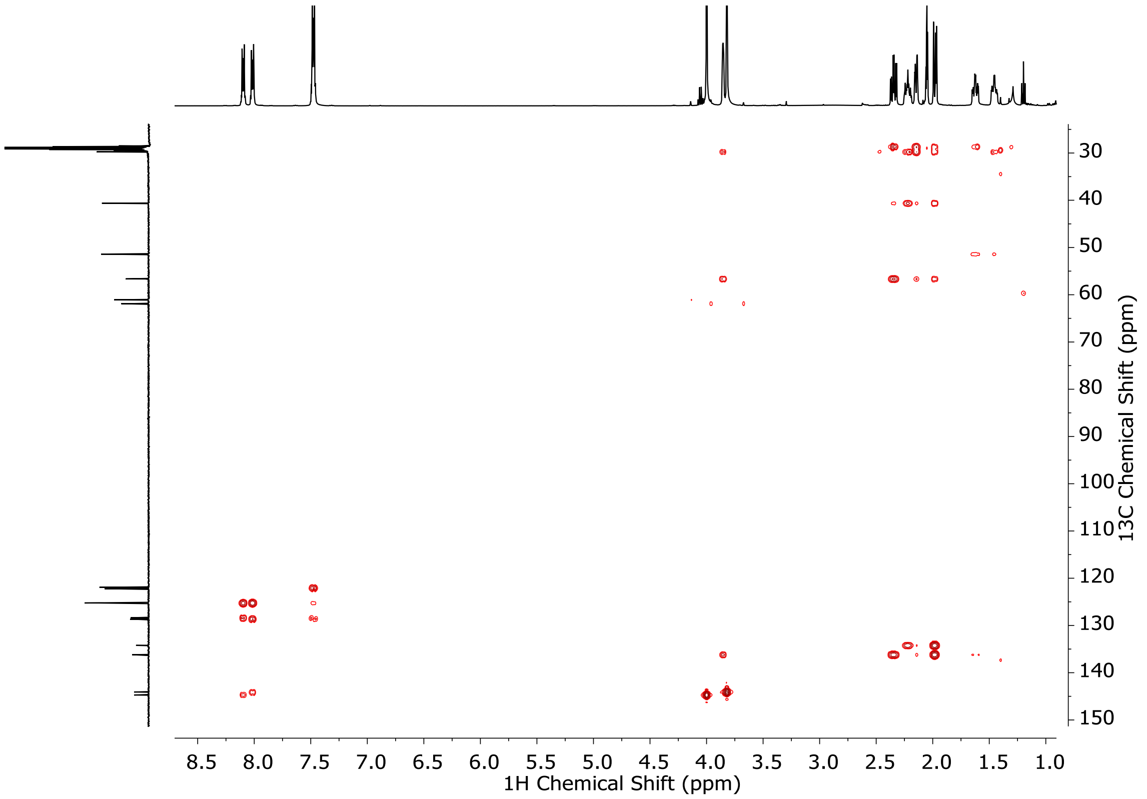

Figure S18. ^{1}H-^{13}C HMBC (500 MHz, Acetone-d_6) spectrum of 9,10-dimethoxy-3,4-dihydro-1,4-methanoanthracene-1(2*H*)-carboxylic acid (**E**).

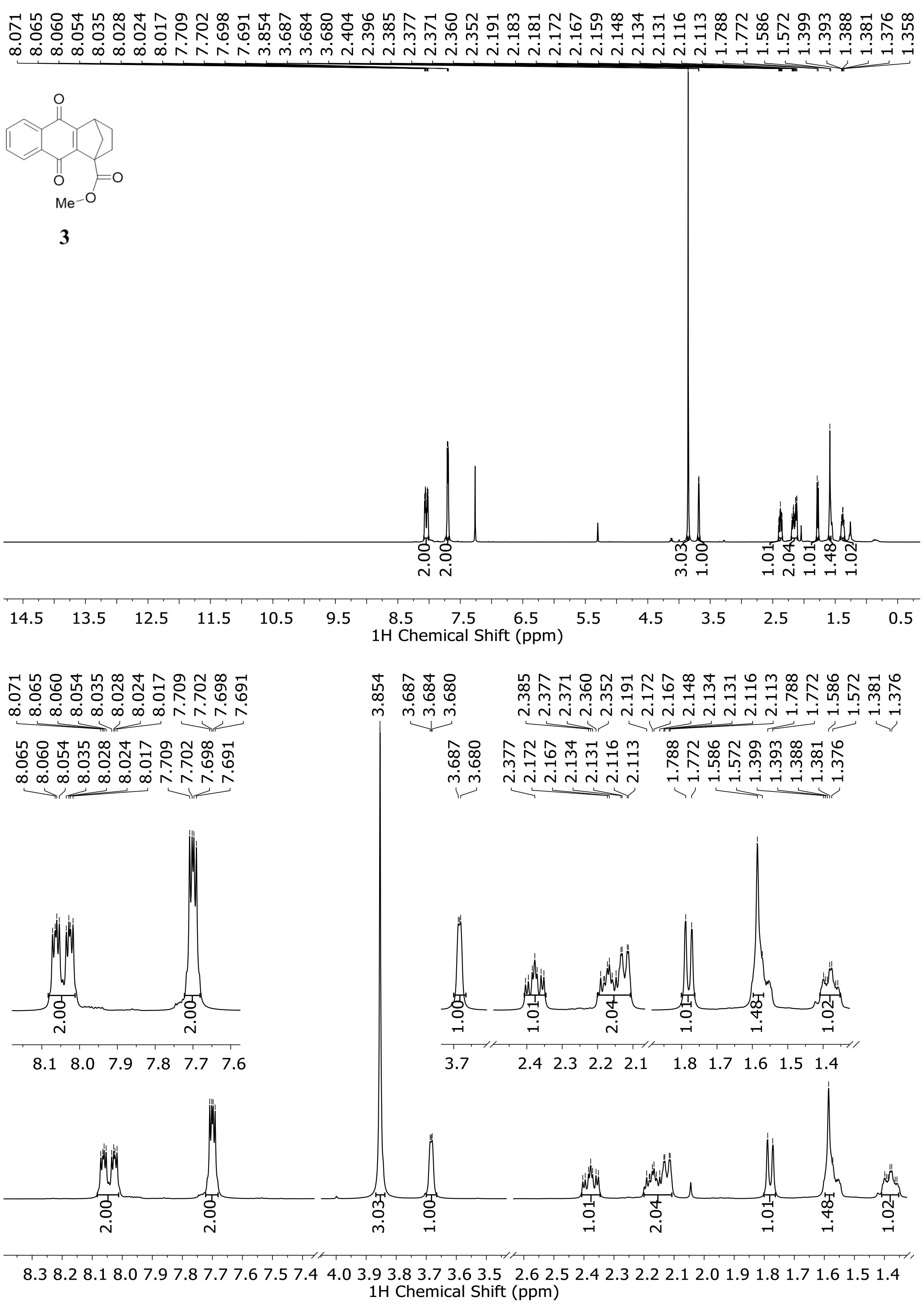

Figure S19. ^{1}H NMR (500 MHz, $CDCl_3$) full spectrum of methyl 9,10-dioxo-3,4,9,10-tetrahydro-1,4-methanoanthracene-1(2*H*)-carboxylate (**3**) (top) and expansion of selected regions (bottom, additional expansions are included in the inset).

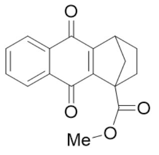

3

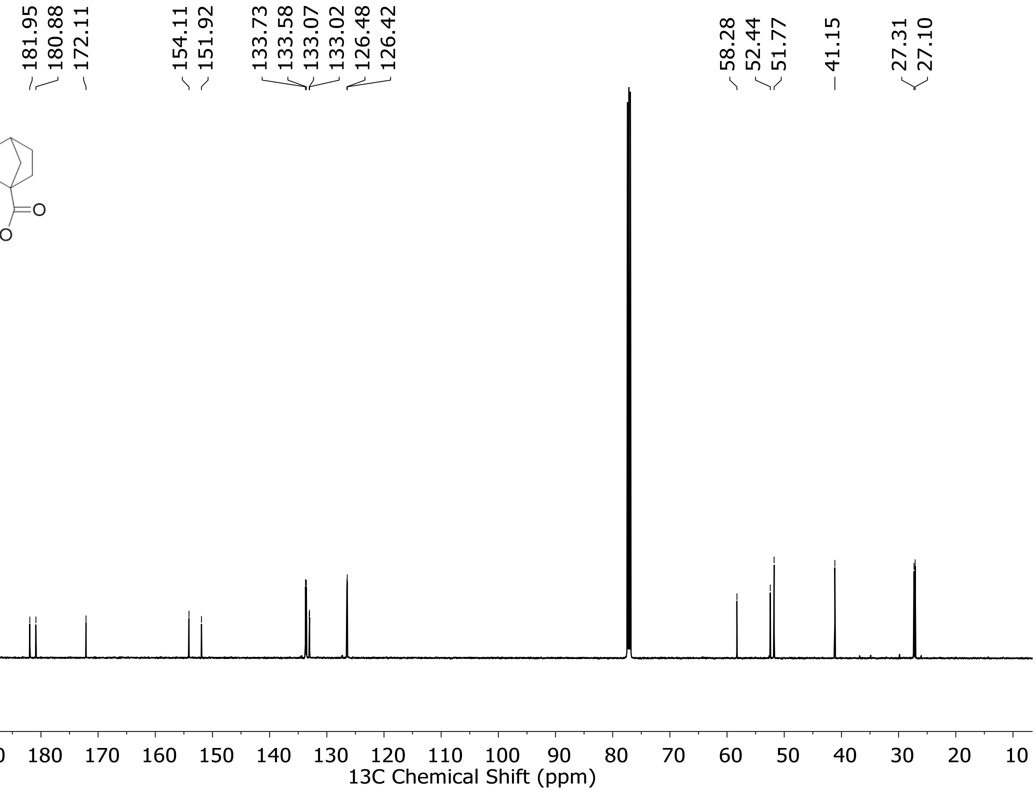

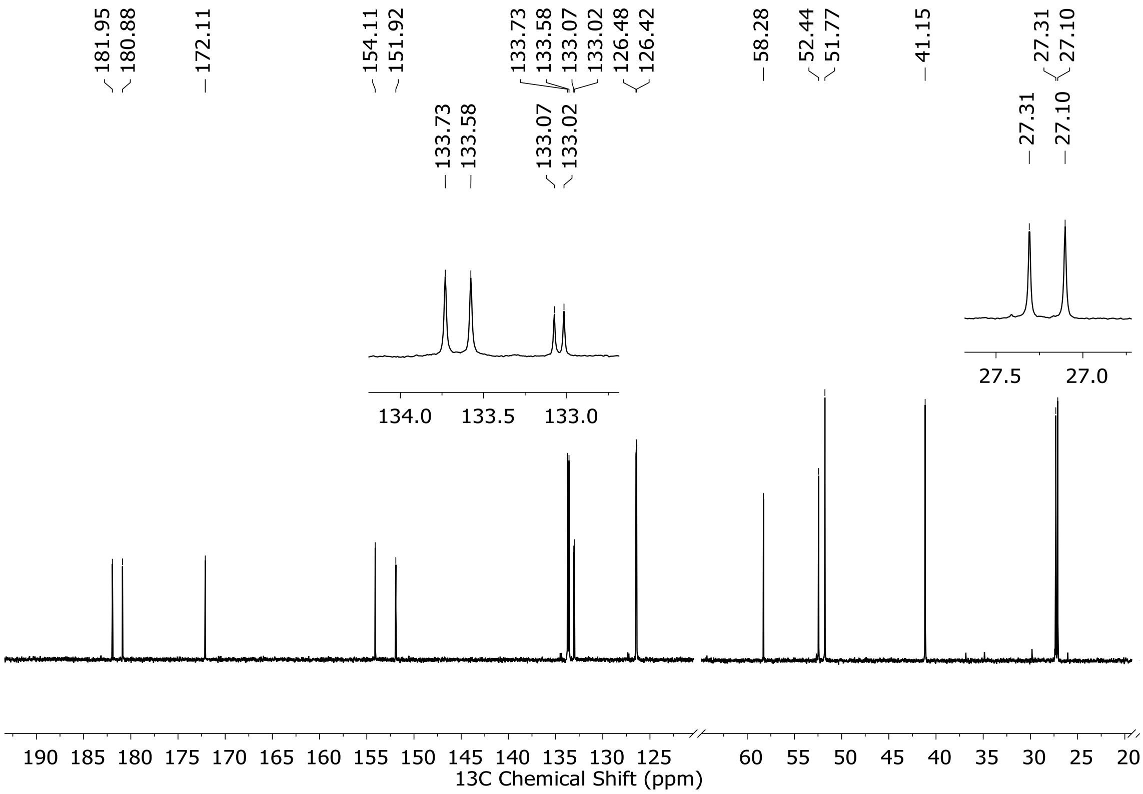

Figure S20. ^{13}C NMR (126 MHz, $CDCl_3$) full spectrum of methyl 9,10-dioxo-3,4,9,10-tetrahydro-1,4-methanoanthracene-1(2*H*)-carboxylate (**3**) (top) and expansion of selected regions (bottom, additional expansions are included in the inset).

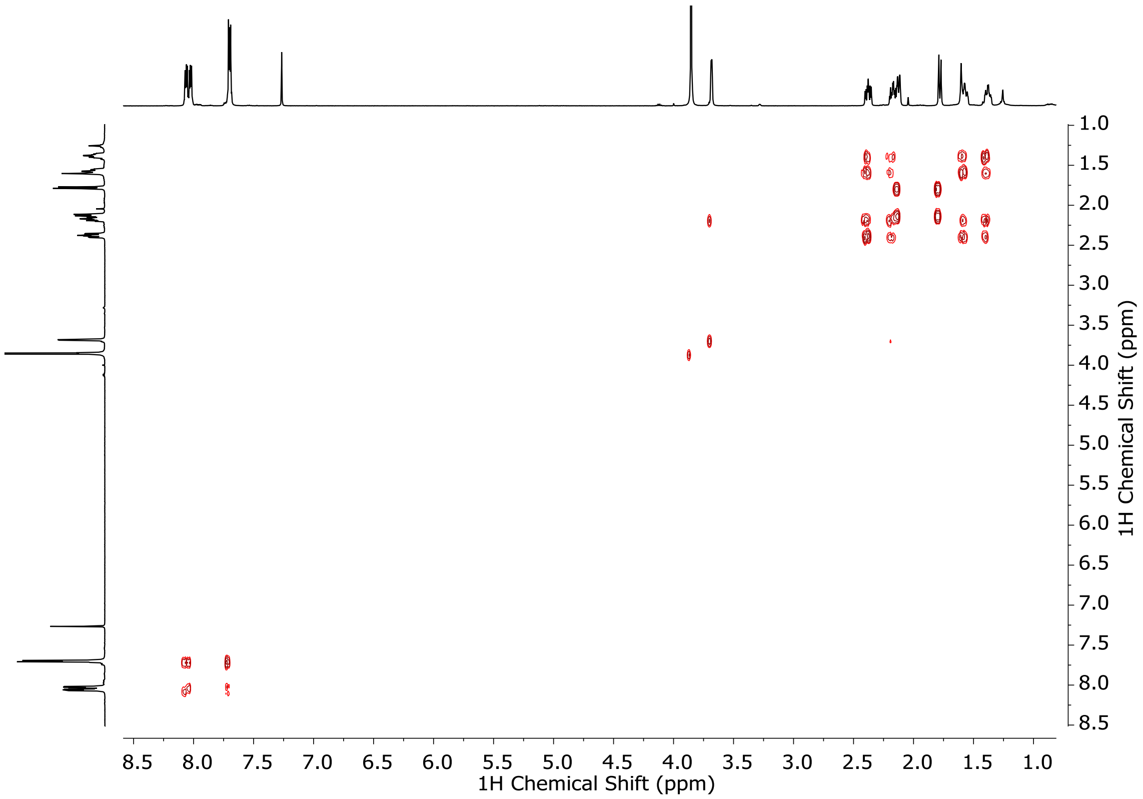

Figure S21. ^{1}H-^{1}H COSY (500 MHz, $CDCl_3$) spectrum of methyl 9,10-dioxo-3,4,9,10-tetrahydro-1,4-methanoanthracene-1(2*H*)-carboxylate (**3**).

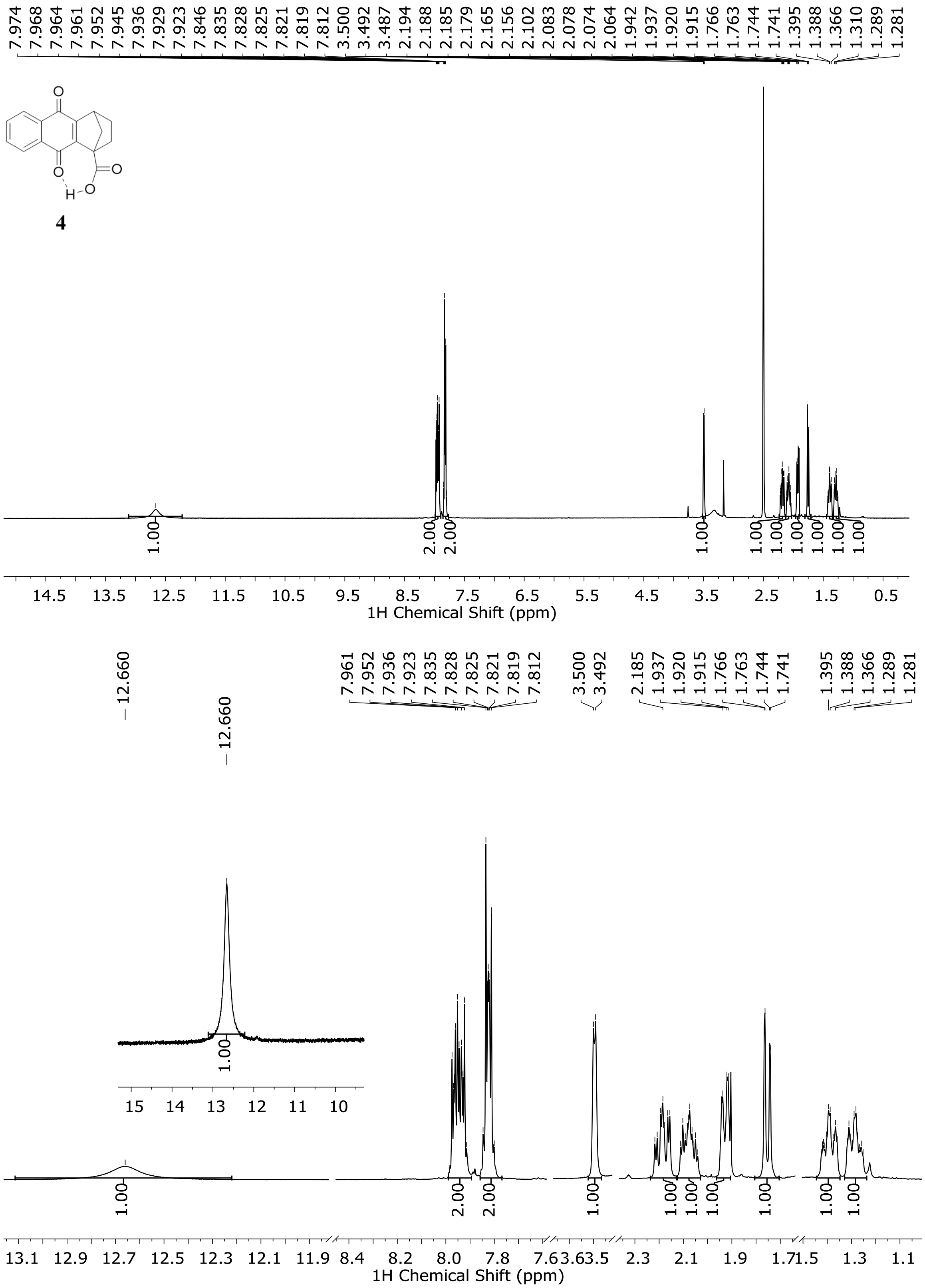

Figure S22. ^{1}H NMR (400 MHz, DMSO-d_6) full spectrum of 9,10-dioxo-3,4,9,10-tetrahydro-1,4-methanoanthracene-1(2*H*)-carboxylic acid (**4**) (top) and expansion of selected regions (bottom, additional expansions are included in the inset).

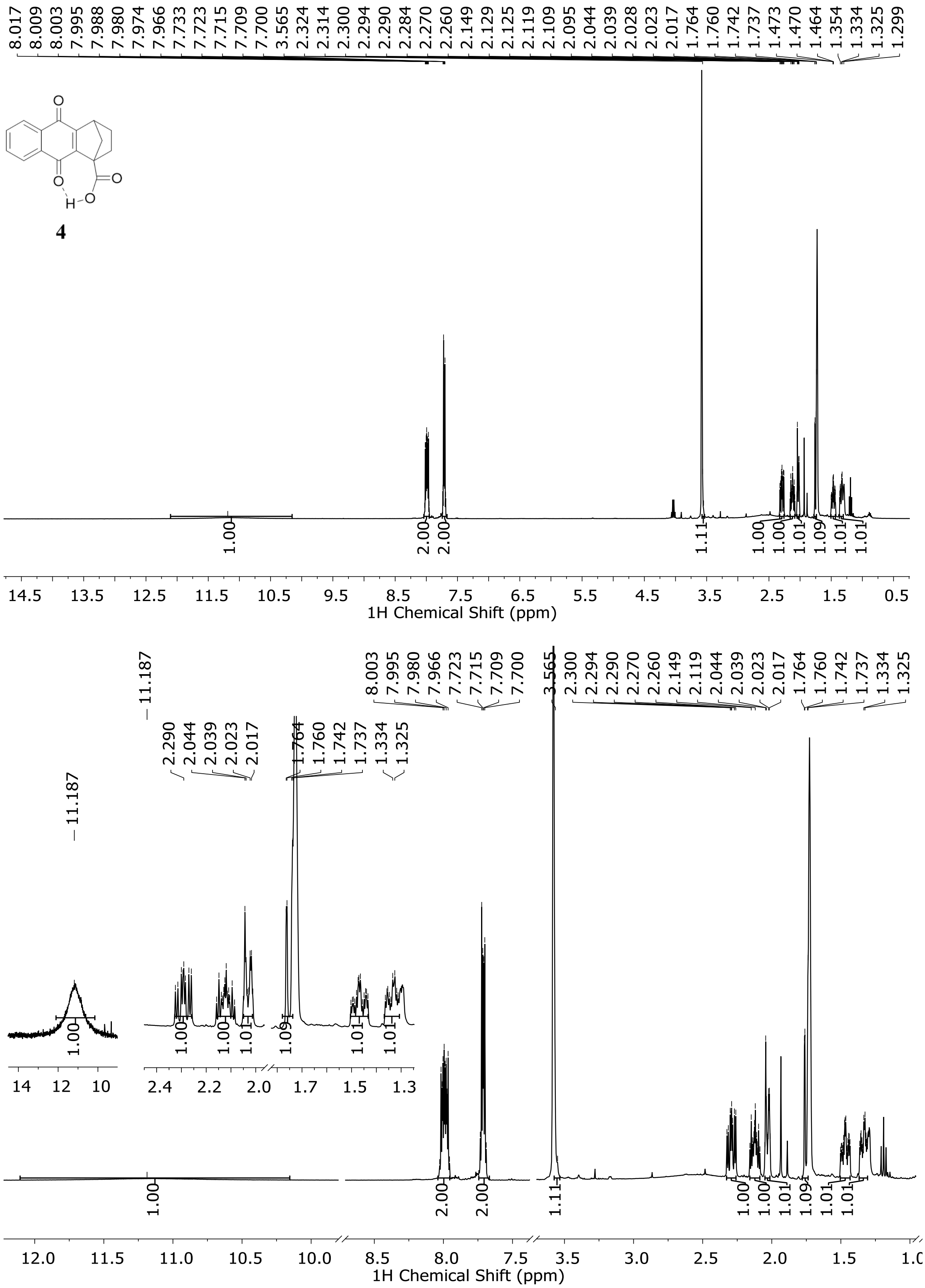

Figure S23. ^{1}H NMR (400 MHz, THF-d_8) full spectrum of 9,10-dioxo-3,4,9,10-tetrahydro-1,4-methanoanthracene-1(2*H*)-carboxylic acid (**4**) (top) and expansion of selected regions (bottom, additional expansions are included in the inset).

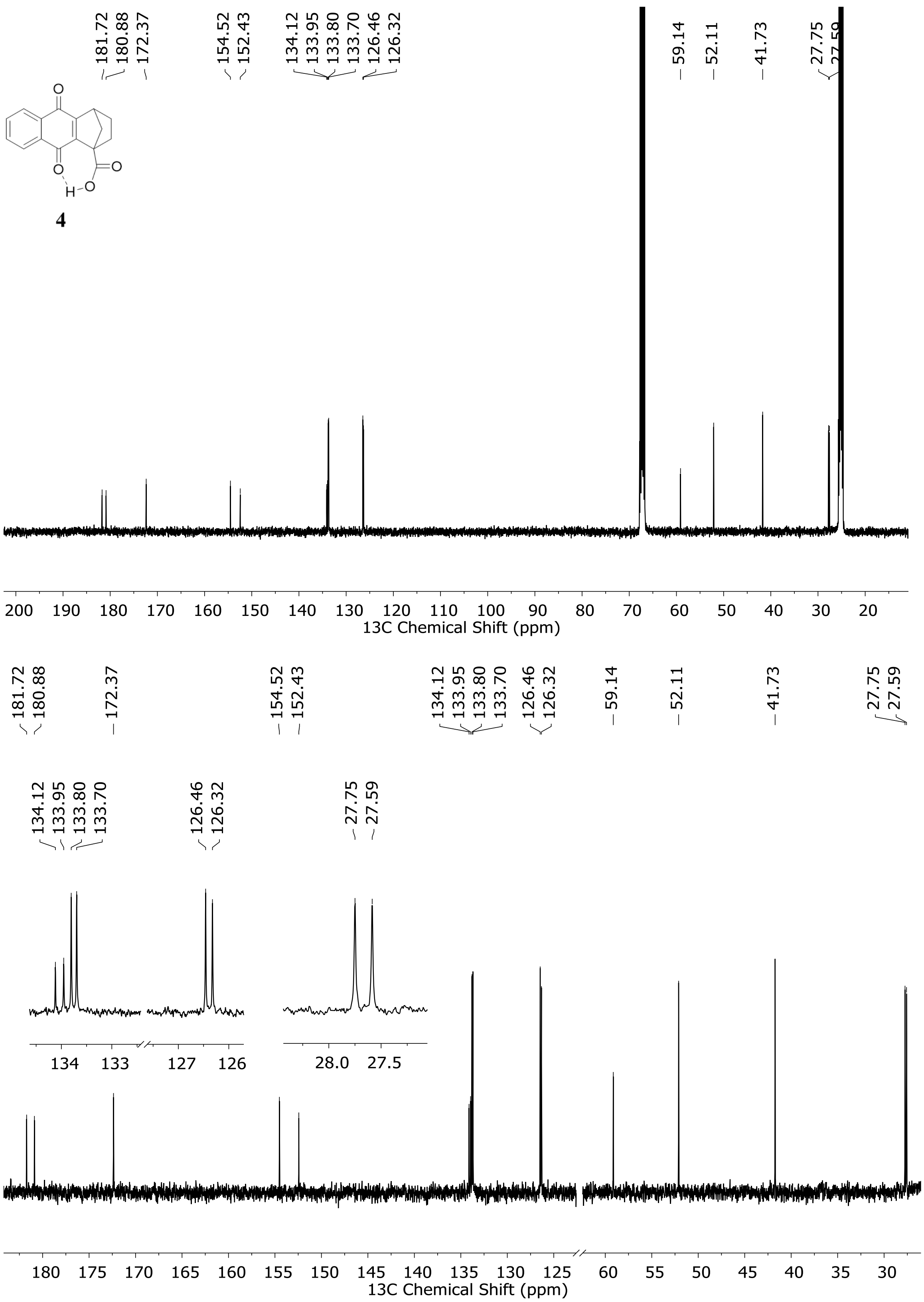

Figure S24. ^{13}C NMR (101 MHz, THF-d_8) full spectrum of 9,10-dioxo-3,4,9,10-tetrahydro-1,4-methanoanthracene-1(2*H*)-carboxylic acid (**4**) (top) and expansion of selected regions (bottom, additional expansions are included in the inset).

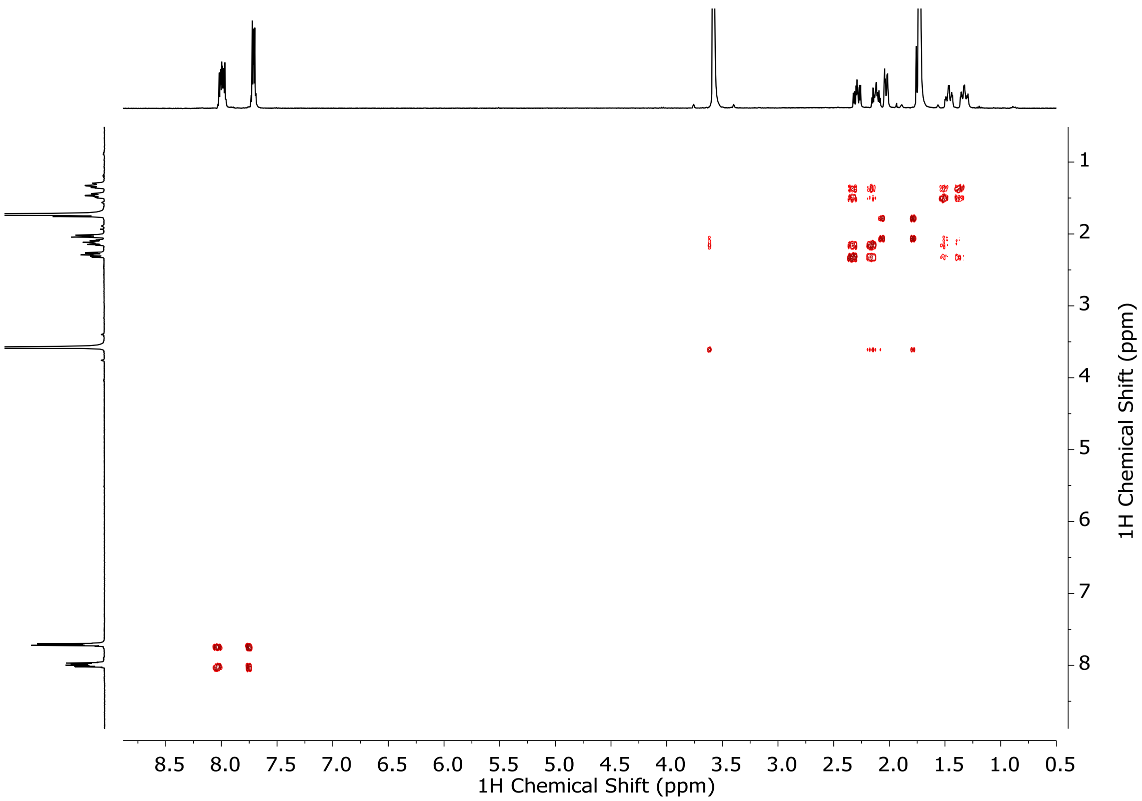

Figure S25. ^{1}H-^{1}H COSY (400 MHz, THF-d_8) spectrum of 9,10-dioxo-3,4,9,10-tetrahydro-1,4-methanoanthracene-1(2*H*)-carboxylic acid (**4**).

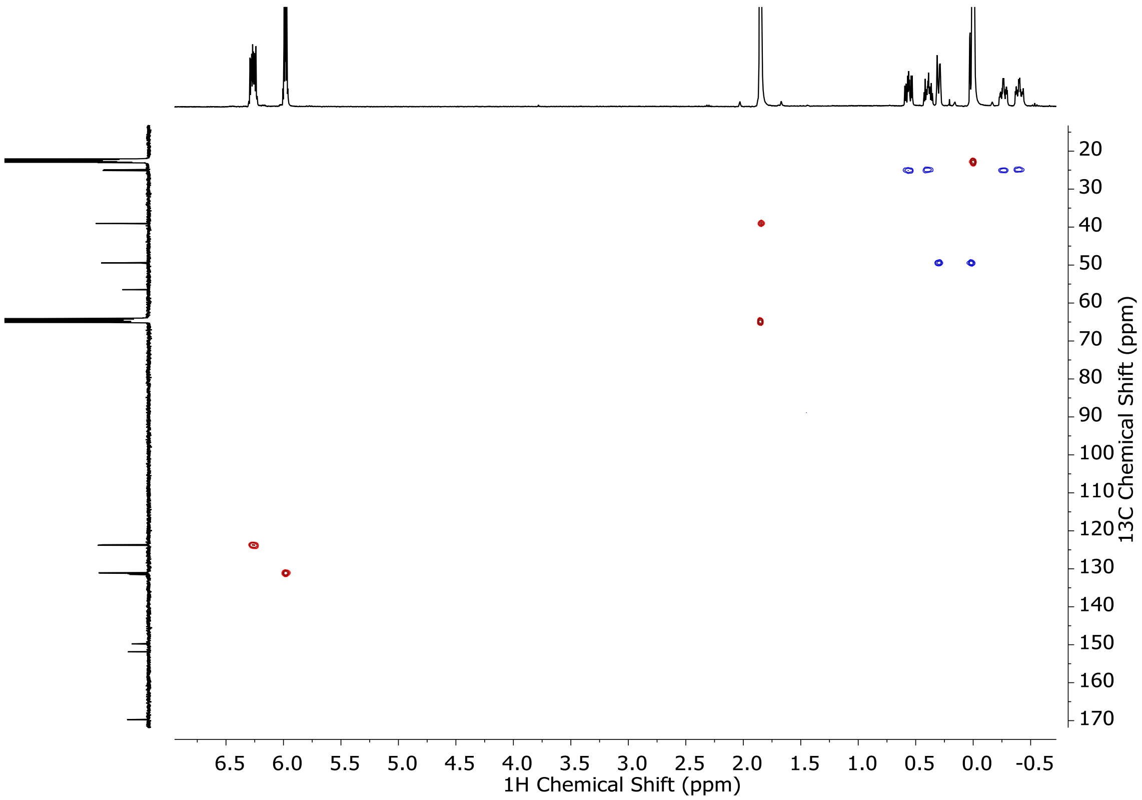

Figure S26. ^{1}H-^{13}C HSQC (400 MHz, THF-d_8) spectrum of 9,10-dioxo-3,4,9,10-tetrahydro-1,4-methanoanthracene-1(2*H*)-carboxylic acid (**4**).

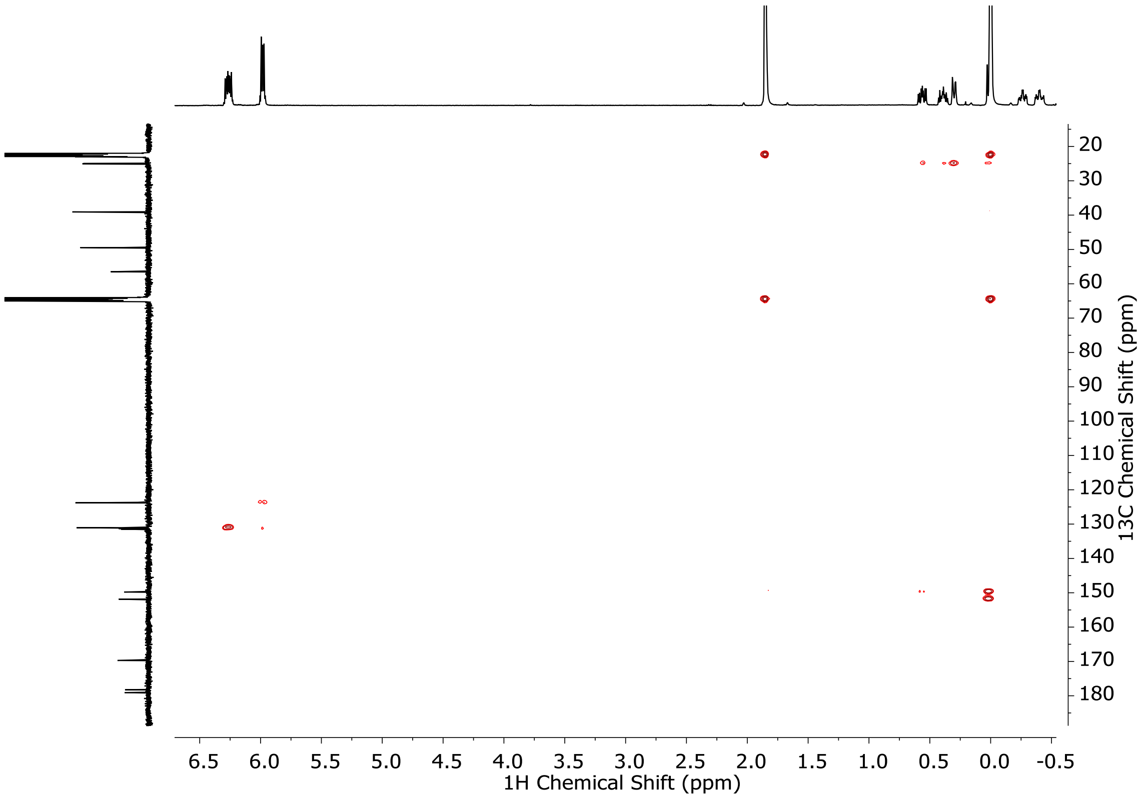

Figure S27. ^{1}H-^{13}C HMBC (400 MHz, THF-d_8) spectrum of 9,10-dioxo-3,4,9,10-tetrahydro-1,4-methanoanthracene-1(2*H*)-carboxylic acid (**4**).

3. Electrochemistry

3.1. Methods: cyclic voltammetry

Cyclic voltammetry measurements were performed with a CH Instruments 760C potentiostat using a glassy carbon (3 mm diameter) working electrode, a Pt wire counter electrode, and a Ag wire pseudoreference electrode in a conventional three-electrode cell. All cyclic voltammograms (CVs) were collected in THF, previously distilled and filtered through a bed of activated basic aluminum oxide and kept over molecular sieves and K_2CO_3, containing the naphthoquinone of interest at a concentration of 1 mM. Tetrabutylammonium hexafluorophosphate ($TBAPF_6$, 0.1 M) was used as the supporting electrolyte, and the scan rate was 100 mV s^{-1}. All measurements were conducted at rt. Solutions were purged with argon to remove oxygen, and argon was passed over the solution during the measurement. The glassy carbon working electrode was polished between measurements with an aluminum slurry on a microcloth polishing pad, followed by solvent rinses, and drying under a stream of nitrogen. The potential of the pseudoreference electrode was determined using the ferrocenium/ferrocene redox couple as an internal standard and adjusting to the saturated calomel electrode (SCE) scale (with $E_{1/2}$ taken to be 0.56 V *vs* SCE in THF).[11] Absolute EtOH (Sigma Aldrich) was added incrementally to the naphthoquinone solution using a microsyringe.

3.2. Cyclic voltammetry of 1 and 3

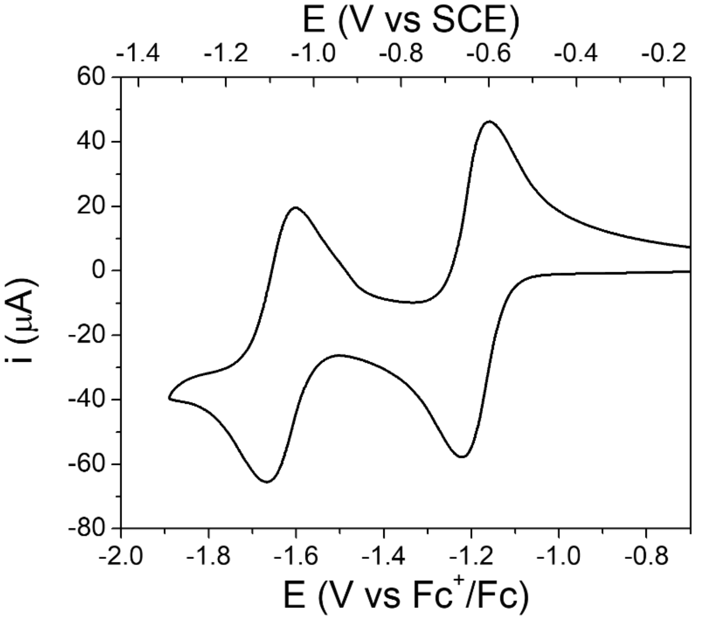

Figure S28. Cyclic voltammogram of **1**. Experimental conditions: 1 mM of **1**, 0.1 M $TBAPF_6$ supporting electrolyte in degassed THF. WE: glassy carbon. Pseudo RE: Ag wire (ferrocene as internal reference). CE: Pt wire. Scan rate, 100 mV s^{-1}.

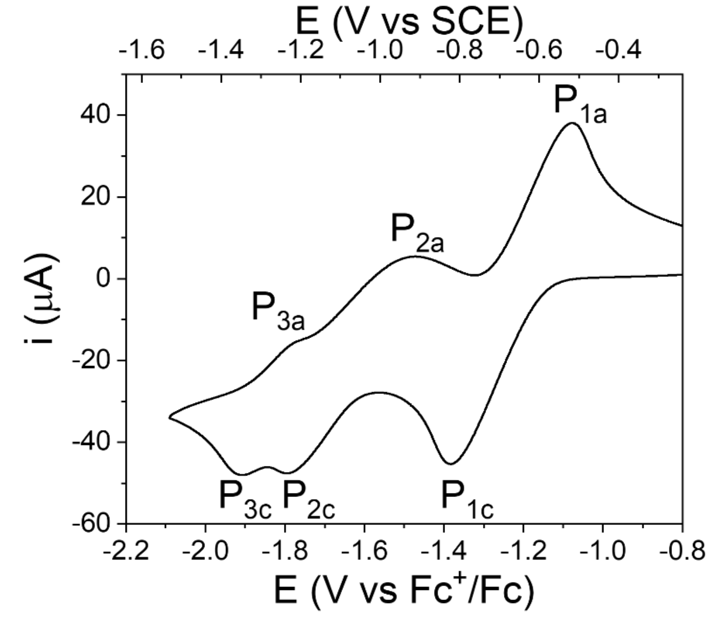

Figure S29. Cyclic voltammogram of **3**. P_{1c} and P_{1a} are the cathodic and anodic peaks, respectively, of the $Q/Q^{•-}$ redox couple, while P_{2c}/P_{3c} and P_{2a}/P_{3a} are the cathodic and anodic peaks, respectively, of the two redox couples in the region of $Q^{•-}/Q^{2-}$. Experimental conditions: 1 mM of **3**, 0.1 M $TBAPF_6$ supporting electrolyte in degassed THF. WE: glassy carbon. Pseudo RE: Ag wire (ferrocene as internal reference). CE: Pt wire. Scan rate, 100 mV s^{-1}.

3.3. Effect of ethanol as weak hydrogen-bonding agent. Mathematical treatment.

The complete mathematical treatment and discussion can be found in the work by Gupta and Henry Linschitz.[12] The quantitative approach considers the hydrogen-bonding constant of the neutral quinone (Q) to be negligible compared to that with mono ($Q^{\bullet-}$) and dianion (Q^{2-}). Accordingly, the following equilibria can be written:

$$Q + e \leftrightharpoons Q^{\bullet-}$$
$$Q^{\bullet-} + nEtOH \leftrightharpoons Q^{\bullet-}(EtOH)_n$$
$$Q^{\bullet-}(EtOH)_n + e \leftrightharpoons Q^{2-}(EtOH)_n$$
$$Q^{2-}(EtOH)_n + (m-n)EtOH \leftrightharpoons Q^{2-}(EtOH)_m$$

By analogy to the treatment made by Peover and Davis's analysis[13] about the dependence of quinone reduction potentials with the concentration and properties of cations, the first reduction step can be expressed as follows:

$$E_{1/2} = E^{\circ}_{1/2} + \left(\frac{RT}{F}\right) ln\left(1 + K_{eq}^{(1)}[EtOH]^n\right) \quad \textbf{(1)}$$

where $E^{\circ}_{1/2}$ is the midpoint potential of the in absence of EtOH, $K_{eq}^{(1)}$ is the association constant of EtOH with $Q^{\bullet-}$ and n denotes the number of EtOH molecules interacting with $Q^{\bullet-}$. If $K_{eq}^{(1)}[EtOH]^n >> 1$, then a plot of $E_{1/2}$ *vs* log[EtOH] should give a straight line with slope 2.3nRT/F, from which n can be estimated (Figure). The values obtained were n = 1.28 and 0.77 for **2** and **4**, respectively. Rearranging eq. 1, $K_{eq}^{(1)}$ can be estimated as follow:

$$exp\left(f\Delta E_{1/2}\right) = 1 + K_{eq}^{(1)}[EtOH]^n \quad \textbf{(2)}$$

where f = F/RT, and $\Delta E_{1/2} = E_{1/2} - E^{\circ}_{1/2}$ (experimental midpoint potentials in the presence and absence of EtOH, respectively, Table S1).

Table S1. Experimental redox potentials for single reduction ($E_{1/2}^{(1)}$) as a function of EtOH addition for **2** and **4**.

[EtOH] (M)	$-E_{1/2}^{(1)}$ of **2** (V vs SCE)	$-E_{1/2}^{(1)}$ of **4** (V vs SCE)
0.07	0.725	0.300
0.10	0.715	0.295
0.14	0.705	0.295
0.17	0.700	0.280
0.21	0.695	0.275
0.24	0.695	0.280
0.27	0.685	0.275
0.31	0.675	0.270
0.34	0.675	0.265

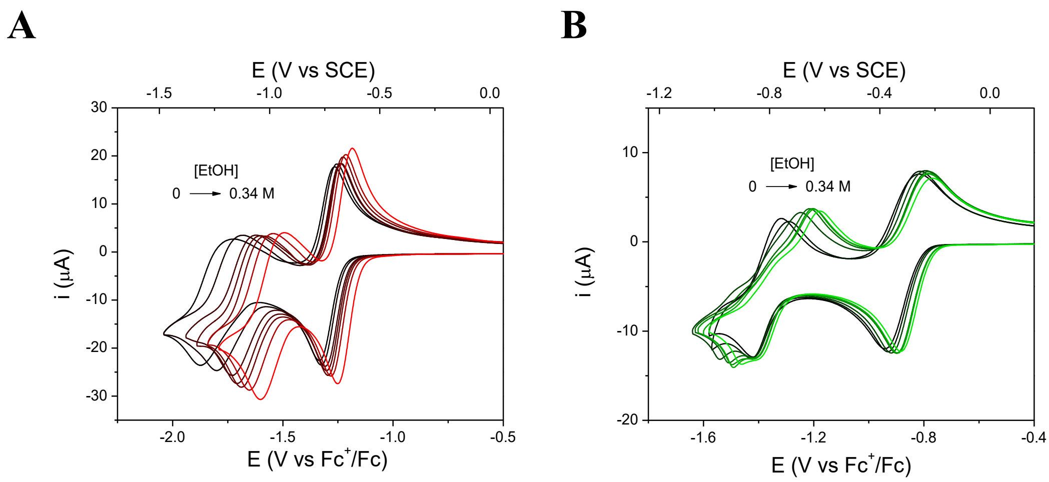

Figure S30. Effect of a weak hydrogen-bonding agent on the reduction processes of **2** and **4**. Cyclic voltammograms of **2** (**A**) and **4** (**B**) at different concentrations of EtOH in THF. Scan rate = 100 mV s^{-1}.

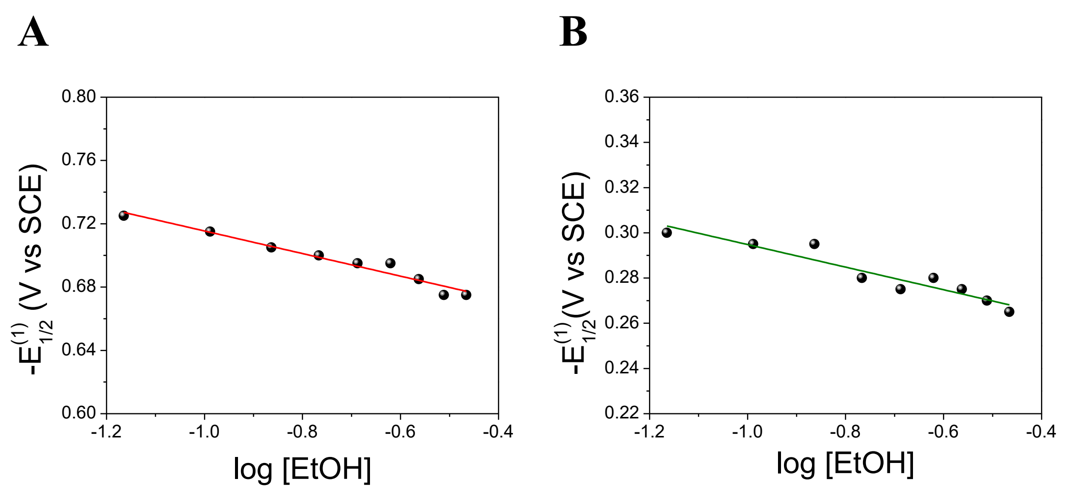

Figure S31. Variation of -$E_{1/2}^{(1)}$ *vs* log [EtOH] in THF for (**A**) **2** and (**B**) **4**. The number of EtOH molecules (*n*) interacting through hydrogen bond can be estimated from the slope of the linear fit.

4. Spectroelectrochemistry (IRSEC) data

The IRSEC measurements were conducted using a Biologic potentiostat connected to an optically transparent thin-layer electrochemical cell (Spectroelectrochemistry Reading RT OTTLE cell), pathlength 0.2 mm, equipped with CaF_2 optical windows. The electrodes arranged in the cell are Pt mesh CE, silver wire pseudo RE, and Pt mesh WE. The WE was positioned in the light path of the IR spectrophotometer. For all the IRSEC measurements, the compounds were used at a concentration of 14 mM in dry THF, and 0.1 M $TBAPF_6$ was used as the supporting electrolyte. A detailed description of the spectroelectrochemical technique is described in a previous report.[14]

5. Infrared (IR) data

FTIR measurements were performed using a Bruker Vertex 70 spectrometer in absorption mode with a 2 cm^{-1} resolution, using a dry nitrogen purge, GloBar MIR source, broadband KBr beamsplitter, and a liquid nitrogen cooled MCT detector. The FTIR experiments were performed using a CaF_2 liquid transmission cell (International Crystal Laboratories, Model SL-3, path length: 0.1030 mm).

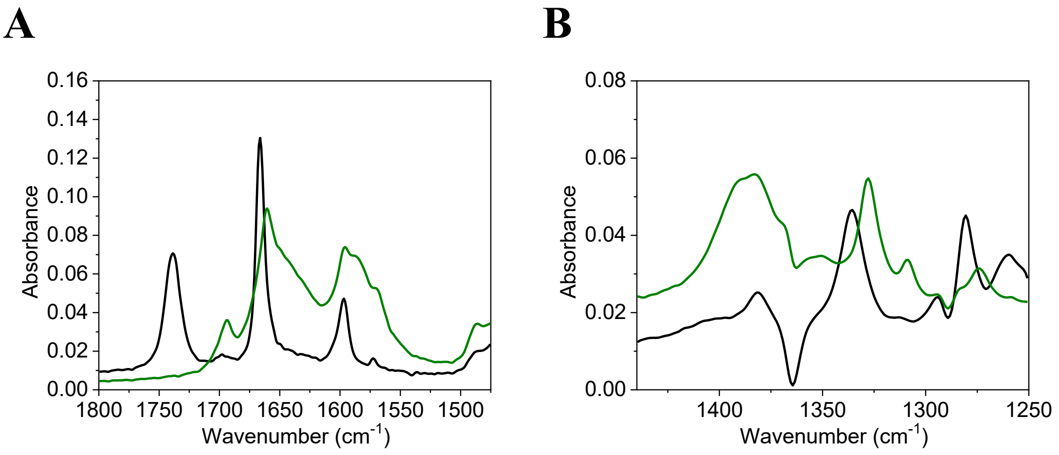

Figure S32. Infrared spectrum of **4** (black line) and after the addition of 5 equivalents of the organic base TBAOH (green line) in the regions 1800 - 1475 cm^{-1} (**A**) and 1450 -1250 cm^{-1} (**B**). Solvent: dry THF.

6. Computational methods

Density functional theory (DFT) calculations using the B3LYP functional[15,16] with dispersion corrections D3BJ[17–19] and the 6-31++G** basis set[20–23] were performed with the Gaussian16 electronic structure program.[24] Geometry optimizations and free energy calculations were performed in THF solution using Bondi atomic radii.[25–27] Solvation effects were included using the conductor-like polarizable continuum model (CPCM),[28,29] and nonelectrostatic contributions of dispersion, repulsion, and cavitation energies were included. This computational protocol has been previously benchmarked on similar systems and shown to yield accurate results.[11,14]

Free energies were calculated using the standard Gibbs relation, $\Delta G^0 = \Delta H^0 - T\Delta S^0$, at T = 298.15 K and include zero-point energy, entropic contributions, and solvation effects. Relative free energies were calculated for all protonation states in both reducing and oxidizing conditions for both the unsubstituted and substituted systems. The redox potentials were computed from the corresponding reaction free energies using an experimentally known reference potential within a thermodynamic cycle. The reference potential used for computing the redox potentials of **1/1**$^{\bullet-}$, **2/2**$^{\bullet-}$, **4/4**$_{\mathbf{A}}^{\bullet-}$, **4/4**$_{\mathbf{B}}^{\bullet-}$ and **4′/4′**$^{\bullet-}$ redox couples (corresponding to the first reduction) was the experimentally measured redox potential of -1.23 V vs Fc^+/Fc in THF of the **3/3**$^{\bullet-}$ redox couple. The reference potential used for computing the redox potentials of the redox couples **1**$^{\bullet-}$**/1**$^{2-}$, **2**$^{\bullet-}$**/2**$^{2-}$, **4**$_{\mathbf{A}}^{\bullet-}$**/4**$^{2-}$, **4**$_{\mathbf{B}}^{\bullet-}$**/4**$^{2-}$ and **4′**$^{\bullet-}$**/4′**$^{2-}$ (corresponding to the second reduction) was the experimentally measured redox potential of -1.64 V vs Fc^+/Fc in THF of the **3**$^{\bullet-}$**/3**$^{2-}$ redox couple. In previous studies,[30] these methods have produced accurate and predictive results in agreement with experiments. The calculated redox potentials are reported in Table 1 in the main text.

By rotating the C–C bond between the carboxylic acid and the NQ backbone, or the C–O bond in $NQH^\bullet$ or NQH^-, we can break or form the intramolecular hydrogen bond. The free energy difference between the conformers with and without an intramolecular hydrogen bond provides an estimate of the hydrogen bond strength. Moreover, the free energy difference between the two protonation states (*i.e.*, the proton on the carboxylate versus the NQ) for the conformers without an intramolecular hydrogen bond provides an estimate of the difference between the covalent O–H bond strength in COOH and NQH. We can thus decompose the shift of redox potentials into contributions from changes in

hydrogen bond strength and changes in covalent O–H bond strength. A full thermodynamic cycle of this decomposition analysis is shown in Figure S33.

To elucidate the vibrational changes upon reduction, the IRSEC spectra of all compounds were simulated for the first and second redox processes (Figure S34 – Figure S36). In particular, simulations of compounds **1–3** served as a reference for modeling the system and identifying specific vibrational modes. To obtain these spectra, normal mode frequency calculations were performed for all compounds in the neutral, single reduced, and double reduced states. For compound **4**, both the $\mathbf{4/4_B^{\bullet-}}$ and $\mathbf{4/4_A^{\bullet-}}$ redox couples as well as the $\mathbf{4_B^{\bullet-}/4^{2-}}$ and $\mathbf{4_A^{\bullet-}/4^{2-}}$ redox couples were modeled. The frequencies were empirically scaled by a factor of 0.983 to match the experimental IRSEC. Specifically, this factor was determined by minimizing the mean square difference of the four peaks shown in Figure 4 in the main text. Frequencies and characterization of selected vibrational modes are given in Table S2 – Table S4. Band assignments were performed by visually inspecting displacements along the computed normal mode vector for each vibration. These computational IRSEC results are in good agreement with the experimentally obtained IRSEC spectra (Figure 4 in the main manuscript).

The molecular electrostatic potentials of compound **4** were calculated using the Gaussian16 electronic structure program.[24] The molecular electrostatic potential (see Eq. 3, main text) calculations were performed for the neutral, single reduced, and double reduced states. The differences between these molecular electrostatic potentials (see Eq. 3, main text) after reduction or oxidation were computed to illustrate the change in electrostatic environment immediately after reduction or oxidation.

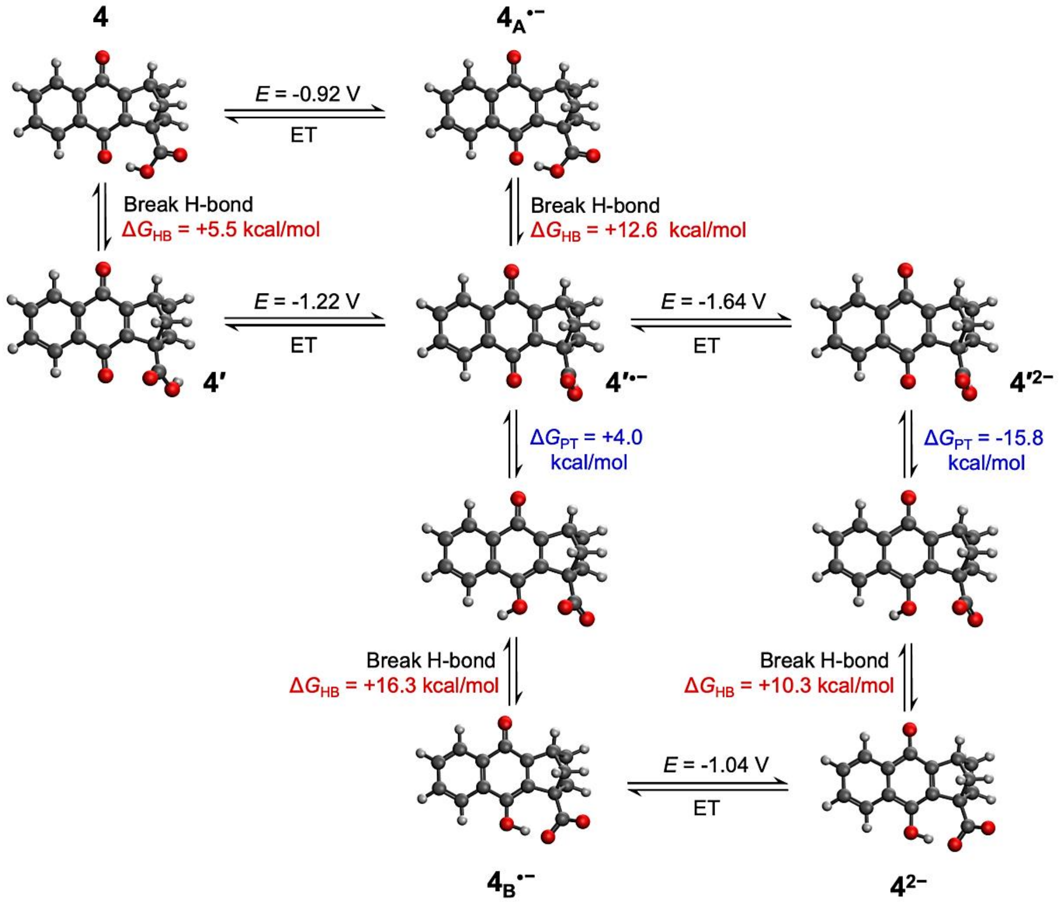

Figure S33. Thermodynamic cycle that decomposes the shift of redox potentials into contributions from changes in hydrogen bond strength and changes in covalent O–H bond strength. The free energies for breaking a hydrogen bond are colored red and are denoted ΔG_{HB}. The free energies for moving the proton from the carboxylic acid to the NQ oxygen for conformers without an intramolecular hydrogen bond are colored blue (and are denoted ΔG_{PT} although they do not correspond to an actual proton transfer event). In **4′**$^{•-}$ and **4′**$^{2-}$, the proton in the carboxylic acid is behind the oxygen and thus is not visible. The redox potentials are given in V vs. $Fc^{+/0}$.

Table S2. Selected peaks and assignments in simulated IRSEC spectra of **1** and **2**.

Compound	Freq.[a]	Peak Description
**1**	1302	NQ[b] breathing
	1604	NQ breathing
	1672	NQ breathing + asymmetric carbonyl stretch
$\mathbf{1}^{\bullet-}$	1442	NQ breathing + asymmetric carbonyl stretch
	1506	NQ breathing + asymmetric carbonyl stretch
$\mathbf{1}^{2-}$	1196	NQ breathing
	1272	NQ breathing + asymmetric carbonyl stretch
	1357	NQ breathing + asymmetric carbonyl stretch
	1527	NQ breathing
**2**	1602	NQ breathing
	1660	NQ breathing + asymmetric carbonyl stretch
$\mathbf{2}^{\bullet-}$	1440	NQ breathing + asymmetric carbonyl stretch
	1508	NQ breathing + asymmetric carbonyl stretch
$\mathbf{2}^{2-}$	1298	C–H bend
	1346	NQ breathing + asymmetric carbonyl stretch
	1561	NQ breathing

[a]Computationally obtained vibrational frequencies scaled by 0.983 reported in cm^{-1}. [b]NQ denotes naphthoquinone.

Table S3. Selected peaks and assignments in simulated IRSEC spectra of **3**.

Compound	Freq.[a]	Peak Description
**3**	1605	NQ[b] breathing + asymmetric carbonyl stretch
	1622	NQ breathing + symmetric carbonyl stretch
	1668	Asymmetric carbonyl stretch + NQ breathing
	1713	Ester C=O stretch
$\mathbf{3}^{\bullet-}$	1446	NQ breathing + asymmetric carbonyl stretch + C–H bend
	1511	Asymmetric carbonyl stretch + NQ breathing + C–H bend
	1695	Ester C=O stretch
$\mathbf{3}^{2-}$	1357	NQ breathing + asymmetric carbonyl stretch + C–H bend
	1562	NQ breathing
	1679	Ester C=O stretch

[a]Computationally obtained vibrational frequencies scaled by 0.983 reported in cm^{-1}. [b]NQ denotes naphthoquinone.

Table S4. Selected peaks and assignments in simulated IRSEC spectra of **4**.

Compound	Freq.[a]	Peak Description
**4**	1450	CA[b] OH bend
	1586,1594	NQ[c] breathing + carbonyl (closer to CA) stretch
	1610	NQ breathing + carbonyl (further from CA) stretch
	1635	Carbonyl (closer to CA) stretch + NQ breathing
	1676	Carbonyl (further from CA) stretch + NQ breathing
	1726	CA asymmetric stretch + OH bend
	2960	OH stretch of CA
$\mathbf{4_A}^{\bullet-}$	1404	NQ breathing + closer carbonyl stretch + OH bend + CA symmetric stretch
	1469	NQ breathing + further carbonyl stretch + OH bend
	1489	NQ breathing + OH bending
	1562	OH bend + CA asymmetric stretch
	1700	OH bend + CA asymmetric stretch
	1845	OH stretch of CA
$\mathbf{4_B}^{\bullet-}$	1341	OH stretch + NQ breathing + C–H bend
	1414	CA asymmetric stretch + closer carbonyl stretch + NQ breathing
	1431	OH bend + NQ breathing
	1467	OH bend + NQ breathing
	1483	OH stretch + NQ breathing + further carbonyl stretch + CA symmetric stretch
	1540	OH bend + CA asymmetric stretch + NQ breathing
	1830	OH stretch
$\mathbf{4}^{2-}$	1368	NQ breathing + OH bend + further carbonyl stretch
	1396	NQ breathing + CA symmetric stretch
	1498,1515	NQ breathing + OH bend + CA asymmetric stretch
	1558	OH bend + CA asymmetric stretch
	2773	OH stretch

[a]Computationally obtained vibrational frequencies scaled by 0.983 reported in cm^{-1}. [b]CA denotes acid. [c]NQ denotes naphthoquinone.

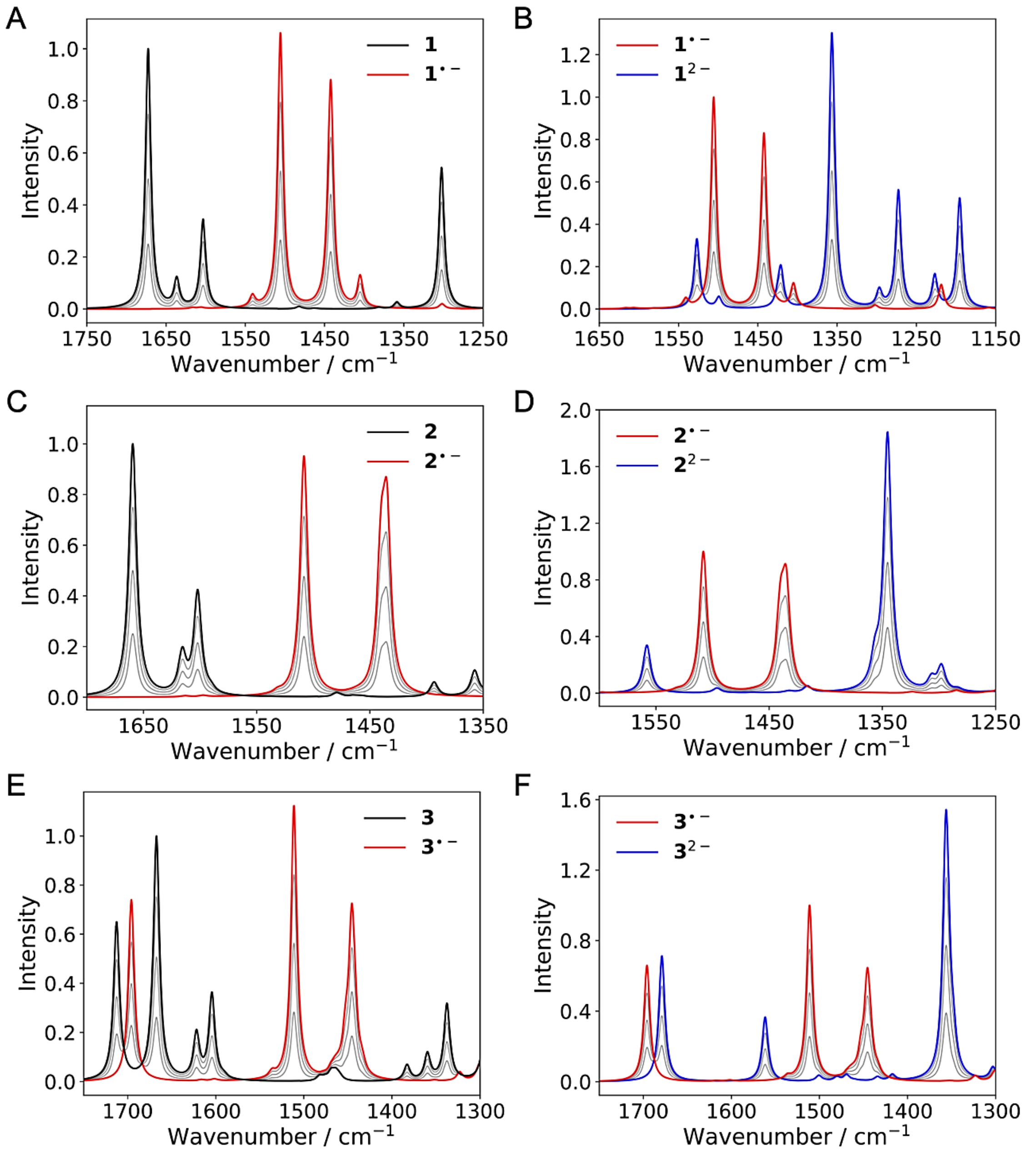

Figure S34. Calculated IRSEC spectra of the first reduction (A, C, E) and the second reduction (B, D, F) of **1**, **2**, and **3**, respectively.

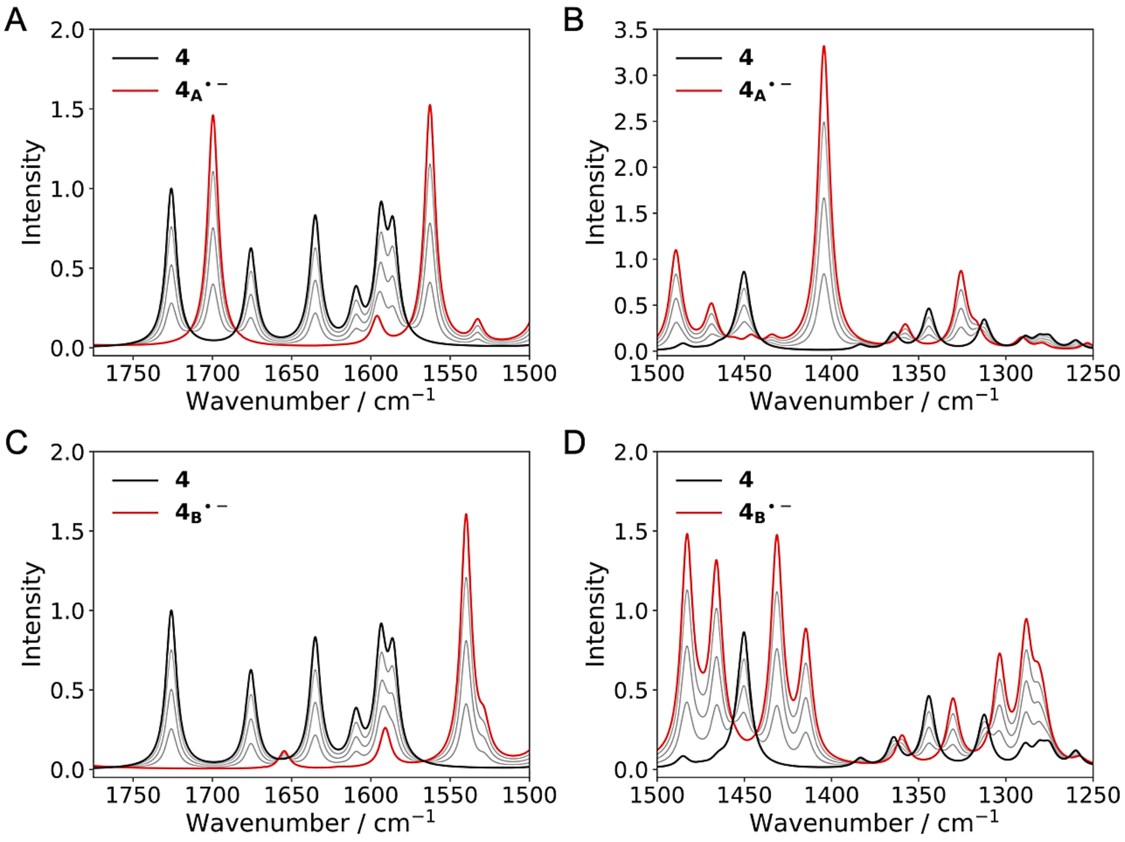

Figure S35. Calculated IRSEC spectra of the first reduction of **4**/**$4_A^{\bullet-}$** (A, B) and **4**/**$4_B^{\bullet-}$** (C, D) at two different spectral regions.

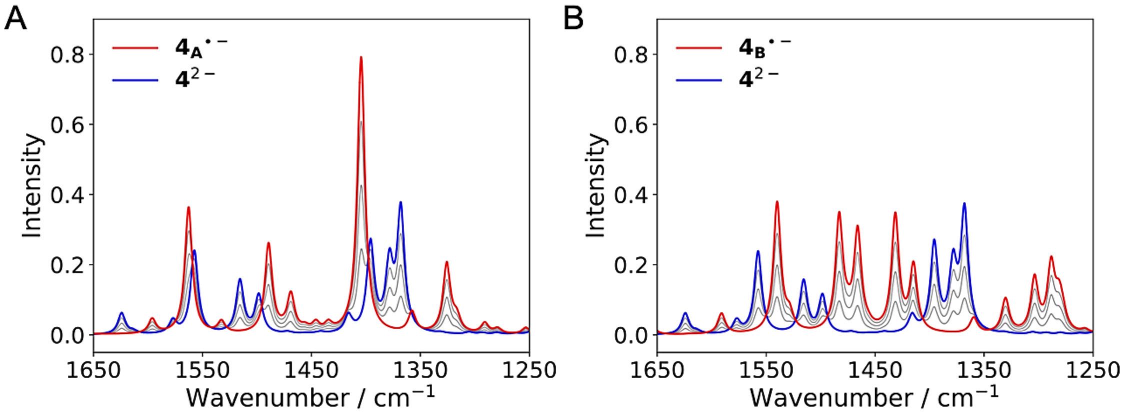

Figure S36. Calculated IRSEC spectra of the second reduction of **$4_A^{\bullet-}$/4^{2-}** (A) and **$4_B^{\bullet-}$/4^{2-}** (B).

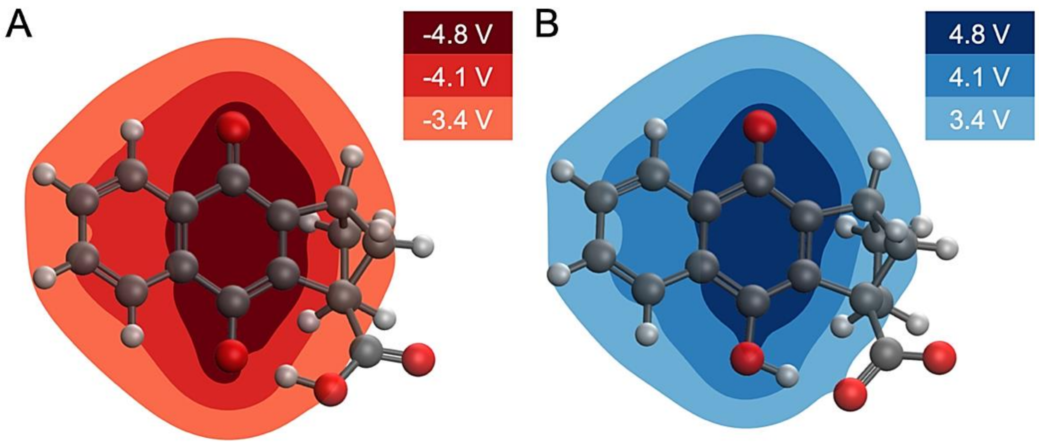

Figure S37. Changes in MEP upon electrochemical reduction or oxidation. (**A**) Change in MEP upon reduction of $\mathbf{4_A}^{\bullet-}$, where proton transfer has not occurred. (**B**) Change in MEP upon oxidation of $\mathbf{4}^{2-}$. The change in MEP is roughly equal and opposite for the reduction and oxidation processes inducing proton translocation. The changes in MEP are given in Volts.

7. References

1 M. Kawamura, S. Kamo, S. Azuma, K. Kubo, T. Sasamori, N. Tokitoh, K. Kuramochi and K. Tsubaki, *Org. Lett.*, 2017, **19**, 301–303.

2 M. S. Akhtar, W.-G. Yang, S. H. Kim and Y. R. Lee, *Asian J. Org. Chem.*, 2021, **10**, 606–613.

3 R. Hongyu and H. Gangliang, *Curr. Org. Synth.*, 2016, **13**, 847–860.

4 M. Gregoritza and F. P. Brandl, *Eur. J. Pharm. Biopharm.*, 2015, **97**, 438–453.

5 G. L. Grunewald and D. P. Davis, *J. Org. Chem.*, 1978, **43**, 3074–3076.

6 D. Gust, T. A. Moore, A. L. Moore, G. Seely, P. Liddell, D. Barrett, L. O. Harding, X. C. Ma, S.-J. Lee and F. Gao, *Tetrahedron*, 1989, **45**, 4867–4891.

7 S. Laugraud, A. Guingant and J. d'Angelo, *Tetrahedron Lett.*, 1989, **30**, 83–86.

8 B. Neises and W. Steglich, *Angew. Chemie Int. Ed. English*, 1978, **17**, 522–524.

9 S. Munawar, A. F. Zahoor, S. M. Hussain, S. Ahmad, A. Mansha, B. Parveen, K. G. Ali and A. Irfan, *Heliyon*, 2024, **10**, e23416.

10 S. Gadigennavar and S. Sankararaman, *Org. Biomol. Chem.*, 2020, **18**, 6738–6744.

11 N. G. Connelly and W. E. Geiger, *Chem. Rev.*, 1996, **96**, 877–910.

12 N. Gupta and H. Linschitz, *J. Am. Chem. Soc.*, 1997, **119**, 6384–6391.

13 M. E. Peover and J. D. Davies, *J. Electroanal. Chem.*, 1963, **6**, 46–53.

14 E. Odella, S. J. Mora, B. L. Wadsworth, M. T. Huynh, J. J. Goings, P. A. Liddell, T. L. Groy, M. Gervaldo, L. E. Sereno, D. Gust, T. A. Moore, G. F. Moore, S. Hammes-Schiffer and A. L. Moore, *J. Am. Chem. Soc.*, 2018, **140**, 15450–15460.

15 C. Lee, W. Yang and R. G. Parr, *Phys. Rev. B*, 1988, **37**, 785–789.

16 A. D. Becke, *J. Chem. Phys.*, 1993, **98**, 5648–5652.

17 P. J. Stephens, F. J. Devlin, C. S. Ashvar, C. F. Chabalowski and M. J. Frisch, *Faraday Discuss.*, 1994, **99**, 103–119.

18 S. Grimme, J. Antony, S. Ehrlich and H. Krieg, *J. Chem. Phys.*, 2010, **132**, 154104.

19 S. Grimme, S. Ehrlich and L. Goerigk, *J. Comput. Chem.*, 2011, **32**, 1456–1465.

20 R. Ditchfield, W. J. Hehre and J. A. Pople, *J. Chem. Phys.*, 1971, **54**, 724–728.

21 W. J. Hehre, R. Ditchfield and J. A. Pople, *J. Chem. Phys.*, 1972, **56**, 2257–2261.

22 P. C. Hariharan and J. A. Pople, *Theor. Chim. Acta*, 1973, **28**, 213–222.

23 M. M. Francl, W. J. Pietro, W. J. Hehre, J. S. Binkley, M. S. Gordon, D. J. DeFrees and J. A. Pople, *J. Chem. Phys.*, 1982, **77**, 3654–3665.

24 M. J. Frisch, G. W. Trucks, H. B. Schlegel, G. E. Scuseria, M. A. Robb, J. R. Cheeseman, G. Scalmani, V. Barone, G. A. Petersson, H. Nakatsuji, X. Li, M. Caricato, A. V. Marenich, J. Bloino, B. G. Janesko, R. Gomperts, B. Mennucci, H. P. Hratchian, J. V Ortiz, A. F. Izmaylov, J. L. Sonnenberg, D. Williams-Young, F. Ding, F. Lipparini, F. Egidi, J. Goings, B. Peng, A. Petrone, T. Henderson, D. Ranasinghe, V. G. Zakrzewski, J. Gao, N. Rega, G. Zheng, W. Liang, M. Hada, M. Ehara, K. Toyota, R. Fukuda, J. Hasegawa, M. Ishida, T. Nakajima, Y. Honda, O. Kitao, H. Nakai, T. Vreven, K.

Throssell, J. A. Montgomery Jr., J. E. Peralta, F. Ogliaro, M. J. Bearpark, J. J. Heyd, E. N. Brothers, K. N. Kudin, V. N. Staroverov, T. A. Keith, R. Kobayashi, J. Normand, K. Raghavachari, A. P. Rendell, J. C. Burant, S. S. Iyengar, J. Tomasi, M. Cossi, J. M. Millam, M. Klene, C. Adamo, R. Cammi, J. W. Ochterski, R. L. Martin, K. Morokuma, O. Farkas, J. B. Foresman and D. J. Fox, 2016.

25 A. Bondi, *J. Phys. Chem.*, 1964, **68**, 441–451.

26 R. A. Pierotti, *Chem. Rev.*, 1976, **76**, 717–726.

27 F. M. Floris J. Tomasi J. L. Pascual Ahuir, *J. Comput. Chem.*, 1991, **12**, 784–791.

28 V. Barone and M. Cossi, *J. Phys. Chem. A*, 1998, **102**, 1995–2001.

29 M. Cossi, N. Rega, G. Scalmani and V. Barone, *J. Comput. Chem.*, 2003, **24**, 669–681.

30 M. T. Huynh, S. J. Mora, M. Villalba, M. E. Tejeda-Ferrari, P. A. Liddell, B. R. Cherry, A. L. Teillout, C. W. MacHan, C. P. Kubiak, D. Gust, T. A. Moore, S. Hammes-Schiffer and A. L. Moore, *ACS Cent. Sci.*, 2017, **3**, 372–380.
